# “Anti‐electrostatic” Halogen Bonding between Ions of Like Charge

**DOI:** 10.1002/chem.202102549

**Published:** 2021-10-01

**Authors:** Jana M. Holthoff, Robert Weiss, Sergiy V. Rosokha, Stefan M. Huber

**Affiliations:** ^1^ Fakultät für Chemie und Biochemie Ruhr-Universität Bochum Universitätsstraße 150 44801 Bochum Germany; ^2^ Institut für Organische Chemie Friedrich-Alexander-Universität Erlangen-Nürnberg Henkestraße 42 91054 Erlangen Germany; ^3^ Department of Chemistry Ball State University Muncie, IN USA

**Keywords:** σ-hole, CSD analysis, DFT calculations, halogen bond, non-covalent interaction

## Abstract

Halogen bonding occurs between molecules featuring Lewis acidic halogen substituents and Lewis bases. It is often rationalized as a predominantly electrostatic interaction and thus interactions between ions of like charge (e. g., of anionic halogen bond donors with halides) seem counter‐intuitive. Herein, we provide an overview on such complexes. First, theoretical studies are described and their findings are compared. Next, experimental evidences are presented in the form of crystal structure database analyses, recent examples of strong “anti‐electrostatic” halogen bonding in crystals, and the observation of such interactions also in solution. We then compare these complexes to select examples of “counter‐intuitive” adducts formed by other interactions, like hydrogen bonding. Finally, we comment on key differences between charge‐transfer and electrostatic polarization.

## Graphical abstract

This article provides an overview on halogen bonding (the noncovalent interaction between Lewis acidic halogen substituents and Lewis bases) between ions of like charge. Next to theoretical studies, experimental examples in the solid state and in solution are covered and a comparison with other interactions is provided.

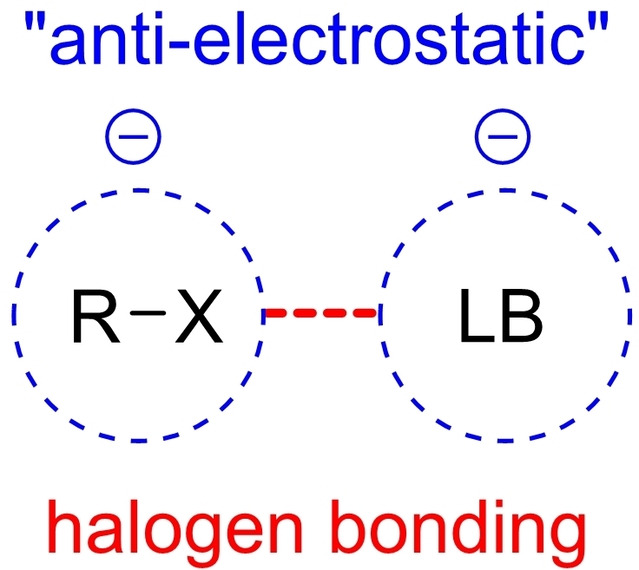

## 1 Introduction

**Figure 1:**
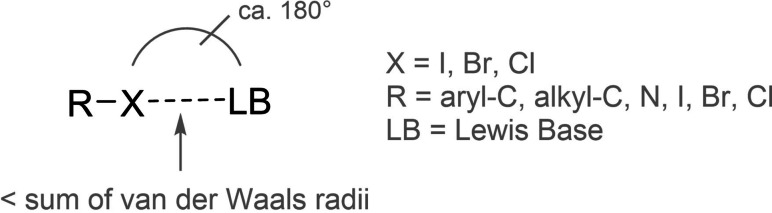
Schematic representation of halogen bonding.

In the last two decades and in particular in the last few years, halogen bonding (XB)[^1^, ^2^] has evolved from the subject of isolated studies to an area of widespread and intense research. This supramolecular interaction between Lewis acidic halogen substituents and Lewis bases (Figure 1) has now found manifold applications in various fields, perhaps most notably in crystal engineering,^[3]^ anion binding,^[4]^ and catalysis.[^5^, ^6^]

These applications take advantage of the specific features of halogen bonding. The strength of this interaction is comparable to the well‐known hydrogen bonding, and it results in reversible formation of supramolecular associations. But in contrast to hydrogen bonding, it features “soft” interacting atoms, such as bromine and iodine. The availability of different halogen substituents also means that there are more options for tuning its strength and the structural features of supramolecular associations, compared to other noncovalent interactions. Next to cationic species, many halogen bond donors (halogen‐based Lewis acids) are based on polyfluorinated arene backbones, and these compounds are often more hydrophobic than comparable hydrogen bond donors. Lastly, and maybe most importantly, halogen bonding is more directional than hydrogen bonding, as the R−X⋅⋅⋅LB angles have to be close to 180° for a reasonable strong attraction to occur.

**Figure 2:**
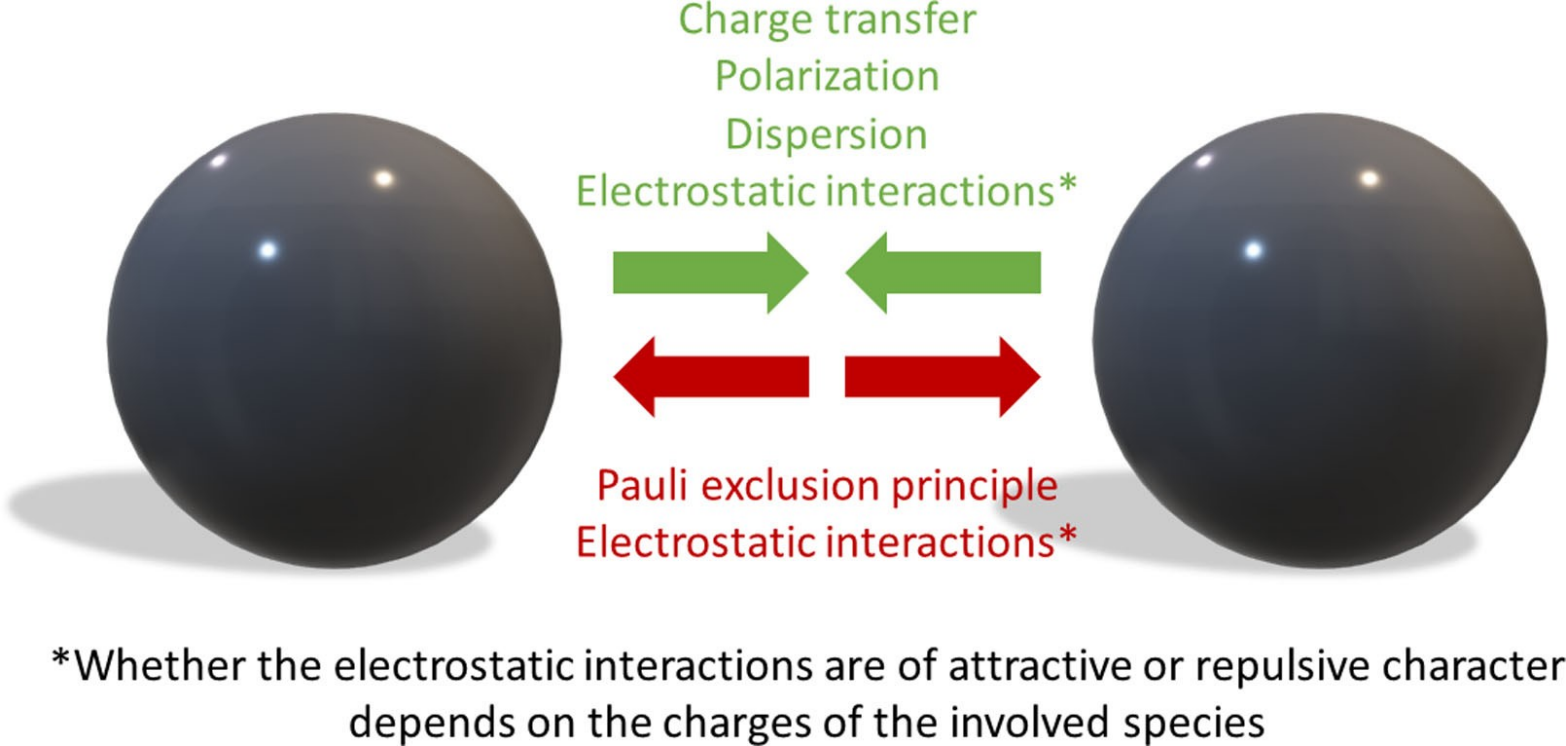
Whether two species attract or repel one another depends on the balance of various attractive (green) and repulsive (red) forces.

The linearity of halogen bonding derives from its electronic origin. The latter is a topic of intense discussions despite the seemingly straightforward fact that, overall, several different attractive and repulsive components could contribute to halogen bonding and need to be considered in its analysis (see Figure 2). It is generally accepted that repulsion may be related to the Coulomb forces (if the interacting partners feature like charges, as is the case for the systems described herein) and to the Pauli exclusion principle (e. g., the repulsion between lone pairs on the halogen and the Lewis base). In comparison, there are ongoing arguments on the importance of dispersion, electrostatics, and charge‐transfer components as bases of the attraction between the interacting partners. It seems clear that the relative magnitudes of the various attractive forces will depend on the specific interaction partners. Weak halogen bonding is likely to be predominantly or mostly dispersion‐driven (to the point where the line between halogen bonding and pure dispersive interactions may be hard to draw). Medium‐strong interactions are apparently dominated by a strong electrostatic component, related to the presence of the so‐called “σ‐hole”, a region of positive potential on the surface of the halogen substituent (opposite the R group). This deformation of electron density has been known from solid state studies, where it was coined “polar flattening”.^[7]^ It should be stressed that electrostatic attraction might be considerably enhanced by the mutual polarization of the binding partners (which substantially alters the “size” of the σ‐hole). Finally, as the interaction becomes stronger and the spheres of the interacting atoms protrude, an increasing amount of charge‐transfer will contribute to the overall binding energy.

**Figure 3:**
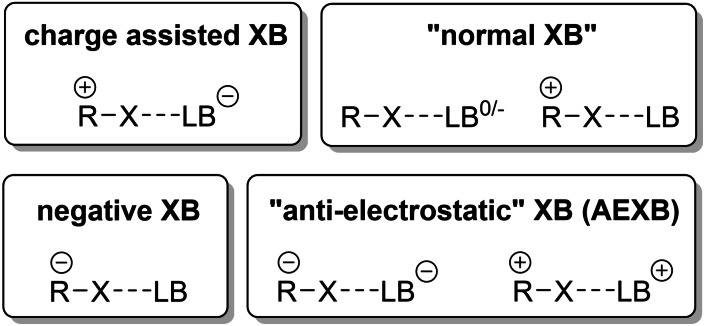
Schematic representation of different kinds of halogen bonding discussed in the literature. In the context of this review, we focus on AEXBs (the attractive interaction between XB donor and acceptor of like charges) as well as so‐called negative XBs (interaction between a negative XB donor and a neutral LB).

The physically clear and visually appealing rationalization of halogen bonding via σ‐hole plots has been very popular in the recent years. This leads in some cases to a purely electrostatic interpretation of this interaction, in which V_S,max_ (the most positive potential on the isosurface of the halogen) has often been used as an indicator for halogen bonding strength. On the other hand, there are also multiple observations[^8^, ^9^, ^10^, ^11^] which point towards the importance of the charge‐transfer contribution for stronger interactions, including trends which run counter to the static σ‐hole and which may be explained by charge‐transfer (even though polarization has also been invoked as rationalization^[12]^). One of the starkest cases which, at first glance, seems to be hard to justify by a purely or predominantly electrostatic reasoning is halogen bonding involving ions of like charges – for instance when anionic halogen bond donors are employed together with negatively charged Lewis bases (Figure 3).

Two notable examples of such complexes have been reported recently,[^13^, ^14^, ^15^] and they have been called “anti‐electrostatic” (AEXBs) to stress this seeming contradiction to a view based purely on electrostatic attraction. (It is important to note that the term was put in quotation marks to indicate the complexity of the attractive forces in these systems, which are affected by the polarization of the interacting species and counterions. Thus, “anti‐electrostatic” is solely meant to indicate a behaviour that is unexpected based on the electrostatic potentials of the isolated binding partners. For hydrogen bonding, the term “counter‐intuitive” was used previously to describe the same phenomenon.^[16]^) While these “unusual” XBs were brought into spotlight lately, an analysis of the literature reveals several examples which could also be classified as AEXBs even though they were not discussed in that context. While many of them apparently involve quite weak interactions, the characteristics of some of the reported associations suggest a noticeable strength of bonding. In general, AEXB is expected to be weaker than interactions based on neutral or positively charged XB donors. However, the identification of such bonding significantly broadens the repertoire of possible XB donors. Besides, the study of AEXB raises awareness for the possibility of such interactions in biochemistry and catalysis. Finally, and maybe most importantly, they provide model cases to investigate the relative contributions of attractive and repulsive components in halogen bonding.

In this concept article, we aim to provide an overview on the known cases of AEXB and will try to put them in perspective in comparison to similar complexes based on other interactions (like hydrogen bonding). We will start with theoretical studies.

## 2 Theoretical Studies

In 2014 Weinhold and Klein started the scientific discourse about the so‐called anti‐electrostatic hydrogen bonding (AEHB). Their theoretical analysis showed that hydrogen bonds between two anionic species might be kinetically stable in vacuum.^[17]^ Nowadays, hydrogen‐bonded associations of two anions are established also experimentally both in the solid state and in solution as described below.

Halogen bonding between ions of like charges was also investigated in silico by various groups. These theoretical studies addressed the (kinetic or thermodynamic) stability of such “anti‐electrostatic” XBs in various environments (gas phase vs. polar solvent). The authors of these works discussed the relevance of individual contributions to the overall binding energies, and, in this context, often stressed the importance of charge transfer. In contrast, a recent paper stressed the Coulombic nature of these interactions.^[18]^

In principle, AEXBs can occur between two (or more) anionic or cationic species. Cationic XB donors are widely used, for example, in the field of crystal engineering or organocatalysis.[^1^, ^2^, ^6^] Usually they form much stronger XBs as compared to their neutral counterparts, especially with anionic Lewis bases.^[19]^ Next to charge assistance, this can also be explained by the increased polarisation of the iodine atom (and a more positive electrostatic potential, that is, a σ‐hole, on its surface) in the cationic species. However, the attractive interaction of a cationic XB donor with a cationic LB (if any) seems counter‐intuitive. Anionic XB donors, on the other hand, typically do not exhibit a region of positive electrostatic potential (ESP) on the halogen atoms. In fact, DFT calculations show that while there are regions of less negative potentials, the ESP values are still negative over the entire surfaces for all examined structures.[^13^, ^15^, ^20^, ^21^, ^22^, ^23^, ^24^] Therefore, the interactions of these XB donors not only with other anions but also with neutral Lewis bases seem unlikely, since two electron‐rich regions must interact for the formation of the respective halogen bonded complexes. Hence, this interaction type involving neutral XB acceptors, called “negative halogen bonding”, is also mentioned in the following as it shares many similarities with AEXBs.

**Figure 4:**
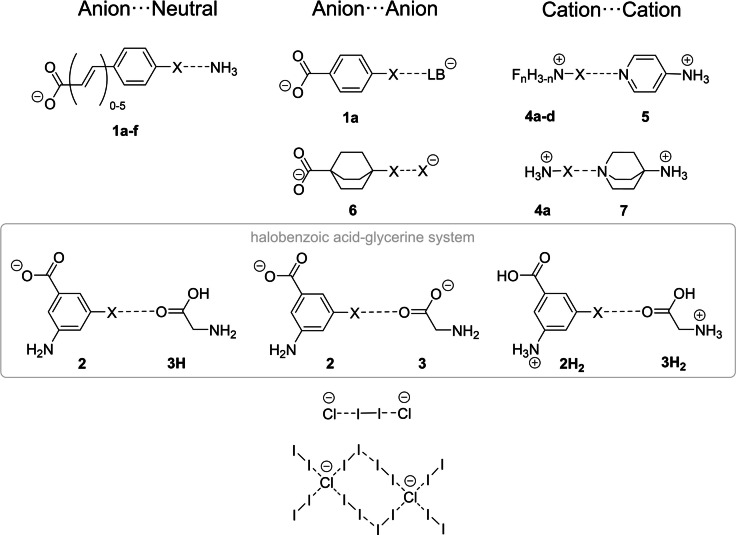
Different types of counter‐intuitive halogen bonding complexes which were examined by means of theoretical studies.[^20^, ^21^, ^22^, ^25^, ^26^, ^27^] (LB=halides or propiolate (HC_2_COO^−^); X=I, Br, Cl, (F)).

Some of the systems in which XB between species with various charges were examined[^20^, ^21^, ^22^, ^25^, ^26^, ^27^] are depicted in Figure 4. In most cases, they are based on derivatives of halobenzoic acid (**1** and **2**), but the inorganic polyhalides [Cl_2_I_2_]^2−^ and [Cl(I_2_)_4_]_2_^2−^ as well as systems like F_n_H_(3‐n)_NX (**4**) or the bicyclic alkane **6** were also considered.

The most comprehensive computational study on AEXBs was reported by Xu, Zhu and co‐workers in 2019.^[27]^ They examined 3‐amino‐5‐halobenzoic acid as XB‐donor in combination with glycine as LB (Figure 4, grey boxed molecules). The charges of these interacting species can be modified by a simple protonation/deprotonation of the acid and amino functionalities without a significant change of the geometries. This allowed the authors to compare and analyse every possible combination of charges in donor and acceptor. Some of the results of the calculations of these systems involving iodinated derivatives are shown in Table 1. The studies of XB with other species led to similar trends and conclusions,^[22]^ which can be summarised as follows:

**Table 1:** Calculated^[a]^ binding energies [kJ⋅mol^−1^] for the 3‐amino‐4‐iodobenzoic acid‐glycerine system (see grey boxed molecules in Figure 4) in different charge states in the gas phase or in polar environment.^[27]^

			charge of		ΔE		
#	complex	X	donor	LB	gas	H_2_O	
1	**2H_2_⋅⋅⋅3**	I	cation	anion	−320	−22.9	charge assisted XB
2	**2⋅⋅⋅3H_2_**	I	anion	cation	−133	−7.0	
3	**2H_2_⋅⋅⋅3H**	I	cation	neutral	−43	−11.4	“normal” XB
4	**2H⋅⋅⋅3**	I	neutral	anion	−64	−14.2	
5	**2H⋅⋅⋅3H**	I	neutral	neutral	−11	−8.3	
6	**2⋅⋅⋅3H**	I	anion	neutral	+6.8	−6.4	negative XB
7	**2H_2_⋅⋅⋅3H_2_**	I	cation	cation	+113	−7.9	AEXB
8	**2⋅⋅⋅3**	I	anion	anion	+132	−9.2	
9	**2⋅⋅⋅3**	Br	anion	anion	+152	−1.7	
10	**2⋅⋅⋅3**	Cl	anion	anion	+158	+2.0

[a] using the M06‐2X functional with the triple‐ζ basis set 6–311++G(d,p) and the Stuttgart/Dresden effective core pseudopotentials and its associated basis set for iodine.

1. The minimum structures which were obtained by a scan of the R−X⋅⋅⋅LB distances display the usual XB geometries, with XB lengths being shorter than the sum of the respective van der Waals (vdW) radii and bond angles close to 180°.[^21^, ^22^, ^23^, ^24^, ^26^, ^27^] This was found for all examined compounds (Figure 4).
2. The binding energies increase in the order Cl<Br<I for the halogen bonding atom (see for example, Table 1, entries 8–10).[^20^, ^21^, ^22^, ^27^] This means that AEXBs and negative XBs follow the same trend (determined by the stronger polarizability of iodine in comparison to bromine or chlorine) as traditional halogen bonding with cationic or neutral donors.^[21]^
3. Calculated AEXB complexes are only kinetically stable and exhibit positive binding energies (ΔE) in the gas phase.[^13^, ^21^, ^24^, ^26^, ^27^] Negative XB complexes, however, sometimes exhibit negative ΔE‐values (e. g., ΔE values of −0.9^[23]^ to −1.3 kJ mol^−1[20]^ were found for the complex **1** **a⋅⋅⋅NH_3_** with X=I). For some examined systems, the calculations did not yield any XB‐bound geometries, indicating that these complexes are not stable in vacuum.[^20^, ^21^] However, in polar environments, the interactions become attractive for almost all investigated combinations.[^13^, ^15^, ^20^, ^21^, ^22^, ^24^, ^26^, ^27^] It is remarkable that according to the results shown in Table 1, AEXBs may be as strong in polar environment as conventional XBs and can even surpass the association strength of a neutral donor with a neutral LB (see entries 5 and 8). Generally, the association strength of AEXB and negative XB complexes increases with increasing dielectric constant.[^20^, ^26^, ^27^]
4. Usually, AEXBs between cationic species are less repulsive/more attractive than the ones between anions (even though in polar environments like water or DMSO, the anion‐anion interaction can also be slightly stronger, see Table 1, entries 7 and 8). This suggests that the XB donor's Lewis acidity plays a more important role than the XB acceptor's Lewis basicity.^[27]^
5. The dissociation barriers of the investigated systems vary significantly. They range from the small ones (≪5 kJ mol^−1^)[^13^, ^22^, ^27^] to values as high as 70 kJ mol^−1^_._ ^[22]^

In addition to the analysis of geometries, binding energies and dissociation barriers, the discussed studies also analysed the origin of the stabilisation of the AEXB complexes.

The overall binding energy can be decomposed into electrostatic (*E*_elstat_), orbital (covalent) (*E*_orb_), dispersion (*E*_disp_) as well as repulsive exchange interaction (*E*_Pauli_) terms. In the case of solvation calculations, additionally solvation energies (*E*_solv_) come into play [Eq. 1].^[27]^

(1) $$E{}_{\mathrm{bind}}=E{}_{\mathrm{elstat}}+E{}_{\mathrm{Pauli}}+E{}_{\mathrm{orb}}+E{}_{\mathrm{disp}}\left(+E{}_{\mathrm{solv}}\right)$$

To elucidate the relative contributions of each term, different energy decomposition and NBO analyses were performed, and single energetic terms (charge transfer or Coulombic interactions) were “switched off” computationally.

NBO analyses of the associations between species in different charge states revealed large charge‐transfer stabilising energies which are similar to those in neutral complexes.^[22]^ The intermolecular transfer of electron density was in all cases directed from the lone pair of the XB acceptor to the σ*‐orbital of the XB donor, regardless of the charges of the involved molecules.[^20^, ^22^, ^23^, ^27^] Zeng et al. concluded that charge‐transfer increases with increasing XB strength and that it is an important factor in the formation of AEXB complexes.^[24]^ Additionally, an intramolecular charge redistribution within the donor and the acceptor (if the latter is not a simple halide anion) is detected. It renders the charge at the halogen atom more positive (or less negative),[^21^, ^22^, ^27^] and can thus attenuate the repulsion between the ions of like charges.^[18]^ This shift of electron density was observed for the aromatic XB donors **1** **a** and **2** as well as for aliphatic compounds like **6**.

**Figure 5:**
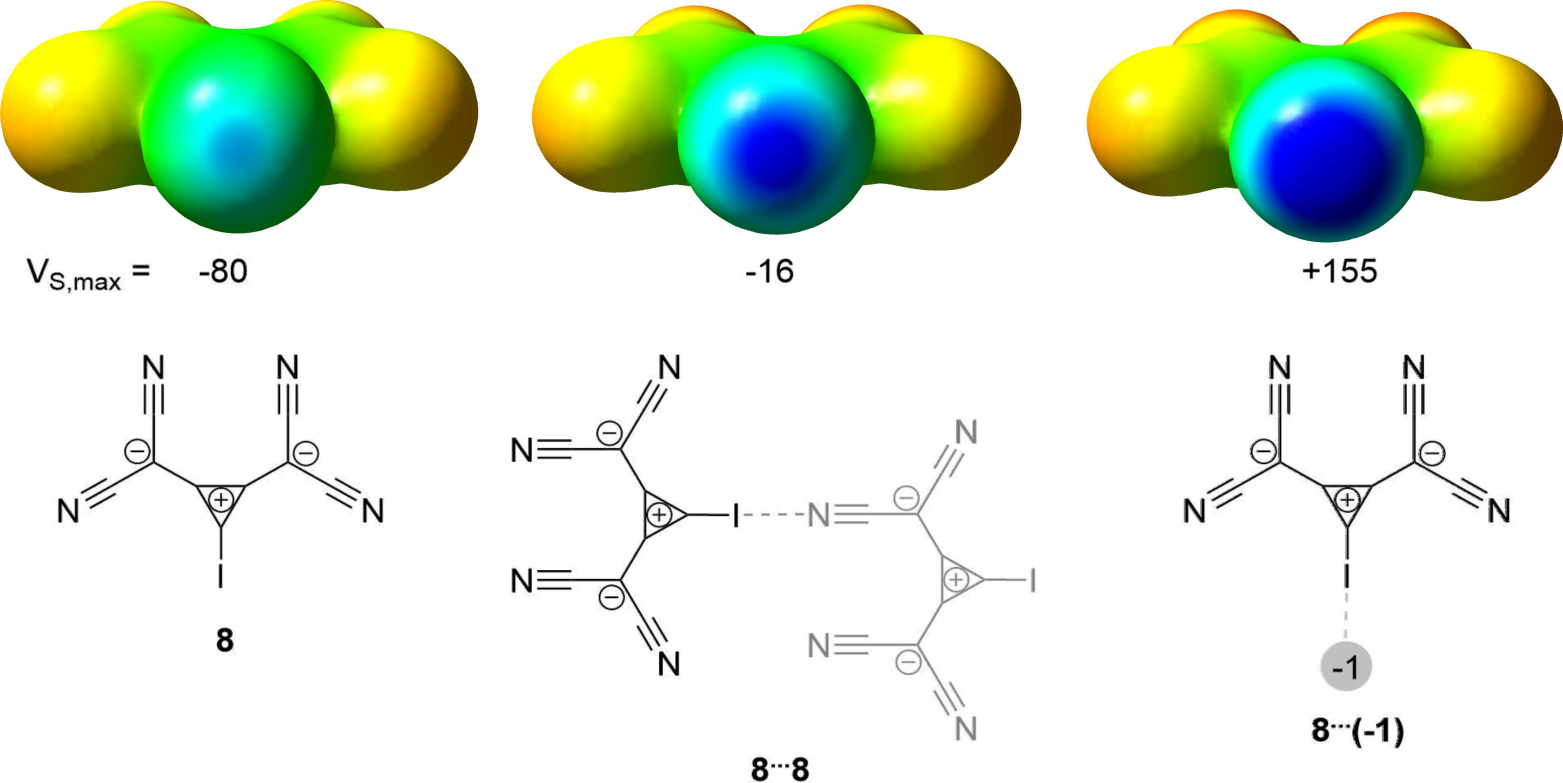
Representation of the calculated electrostatic potential on the 0.001 au isosurface of 1,2‐bis(dicyanomethylene)‐3‐iodo‐cyclopropanide **8** with (middle and right) and without (left) point charges (M06‐2X/def2‐TZVP with an additional diffuse function for iodine).^[13]^ The scale ranges from −454 (red) to 0 kJ mol^−1^ (blue). Moreover, the local electrostatic potential maxima (V_S,max_) in kJ mol^−1^ at the iodine atoms are given.

An analogous effect of mutual polarization of interacting species was identified in the recently published examples of AEXBs involving the anionic 1,2‐bis(dicyanomethylene)‐3‐iodo‐cyclopropanide **8** ^[13]^ (see Figure 5) and tetraiodo‐*p*‐benzoquinone radical **17** ^[15]^ in the solid state. Using the “point‐charge approach”,[^11^, ^28^] in which an anionic 1e charge is positioned in the proximity to the halogen atom of the donor, the authors showed that the presence of the electron‐rich LB in the vicinity of the XB donor causes a substantial polarization of the halogen atom. This results in a significant change in the magnitude of the ESP associated with the σ‐hole (see for example Figure 5).[^13^, ^15^]

For example, the potential on the surface of the iodine atom changes from −80 kJ mol^−1^ in the isolated anion **8** to 155 kJ mol^−1^ in the presence of 1e negative charge.^[13]^ In a similar way, the negative σ‐hole potential of −150 kJ⋅mol^−^1 on the surface of the iodine atom in the isolated radical anion tetraiodo‐*p*‐benzoquinone changes to a positive +130 kJ mol^−1^ value in the presence of the 1e negative point charge.^[15]^ It should be mentioned, however, that the analysis of the self‐associated XB donor **8** revealed a somewhat different picture (such motifs were also identified in the solid‐state studies). To calculate the ESP values on the surface of the XB donor in such associations, the XB acceptor (Figure 5, grey molecule) was replaced with the atomic point charges located at the positions of each atom. In the presence of these charges, the magnitude of the negative potential on the surface of the iodine atom in the anionic XB donor decreased as compared to the initial value (in the isolated anion **8**). However, it remained negative.^[13]^ These results suggest that although polarisation appears to be an important factor in the formation of AEXBs, other terms might also be of high relevance.

To examine the electronic components of AEXB, as well as in the bonding of two inorganic systems, [Cl_2_I_2_]^2−^ and [Cl(I_2_)_4_]_2_^2−^, Wang, Shaik, Mo and co‐workers used the block‐localised wavefunction (BLW) method, which allows one to eliminate the charge transfer (CT) contributions in the binding between two partners.^[17]^ They found that the hydrogen‐bonded systems with quenched CT interaction were still kinetically stable, but the kinetic stabilisation of the complexes was substantially reduced. For the polyhalides, the calculations demonstrated that the CT contribution is the most important energy component.

Xu, Zhu and co‐workers used the so‐called “free‐radical” approach to examine the XB components in complexes between differently charged derivatives of halobenzoic acid and glycine.^[25]^ They found that after elimination of the background Coulombic interaction, the binding energies in the XB complexes were almost always negative (i. e. they remained stable). Moreover, the differently charged substituents in the interacting species still influenced the binding energies.

The components of the binding energies in the AEXB or negative XB complexes were also studied using different energy decomposition analyses. In particular, the relevant forces for the negative XBs of **1** **a**–**f** with ammonia and in the negative and anti‐electrostatic halogen bonding in the halobenzoic acid‐glycerine systems were examined using the ETS‐NOCV scheme within the Amsterdam Density Functional (ADF) program.[^23^, ^27^] The interaction in the **1** **a**⋅⋅⋅X^−^ complex and in systems with aliphatic donors like **6** were explored using the SAPT (symmetry adapted perturbation theory) method.[^21^, ^22^] These studies revealed that the electrostatic energy terms are positive (i. e., their contribution is repulsive) for most of the negative and AEXBs associations,[^13^, ^14^, ^19^, ^23^] although a few examples of attractive electrostatic interaction were found for cation⋅⋅⋅cation AEXBs such as **4** **a**⋅⋅⋅**7** (see Figure 4),^[22]^ as well as for negative XBs (anionic donor⋅⋅⋅neutral LB) and AEXB.^[27]^ Elguero et al. examined the components of the interaction energies in minima XB structures and in corresponding transition states (structure at longer distances than the minimum structure exhibiting a local maximum in ΔE). They found that the electrostatic term is always more repulsive in the transition state than for the minima.^[22]^

In contrast to the electrostatic component, the energy decomposition analyses showed that dispersion and orbital interactions were always attractive.[^23^, ^27^] While the magnitude of the latter substantially depends on the charge of the interacting species, the dispersion term is independent of the charges and the environment.[^23^, ^27^] Although it is usually relatively small, it is non‐negligible for weak AEXBs.^[27]^ The covalent contribution *E*_orb_ is the most important attractive energy term according to the analyses of the halobenzoic acid/glycine system,^[27]^ and for other complexes such as **1** **a**⋅⋅⋅[HC_2_COO]^−^ (see Figure 4) the importance of inductive terms is stressed.[^21^, ^22^]

The above‐presented seven theoretical studies demonstrated that AEXB complexes can be stable under the right conditions. Polar environments seem to reduce the Coulomb repulsion between the binding partners so that the majority of the investigated systems showed negative binding energies when solvation models were applied. Moreover, counterions (which were not considered in these studies) could also have a crucial stabilising effect, predominantly in the solid state. Nevertheless, these works suggest that orbital interactions and inductive/polarisation effects represent probably the most important energetic contributions necessary to overcome the electrostatic repulsion in the minimum structures.

## 3 AEXB in the Solid State and in Solution

**Figure 6:**
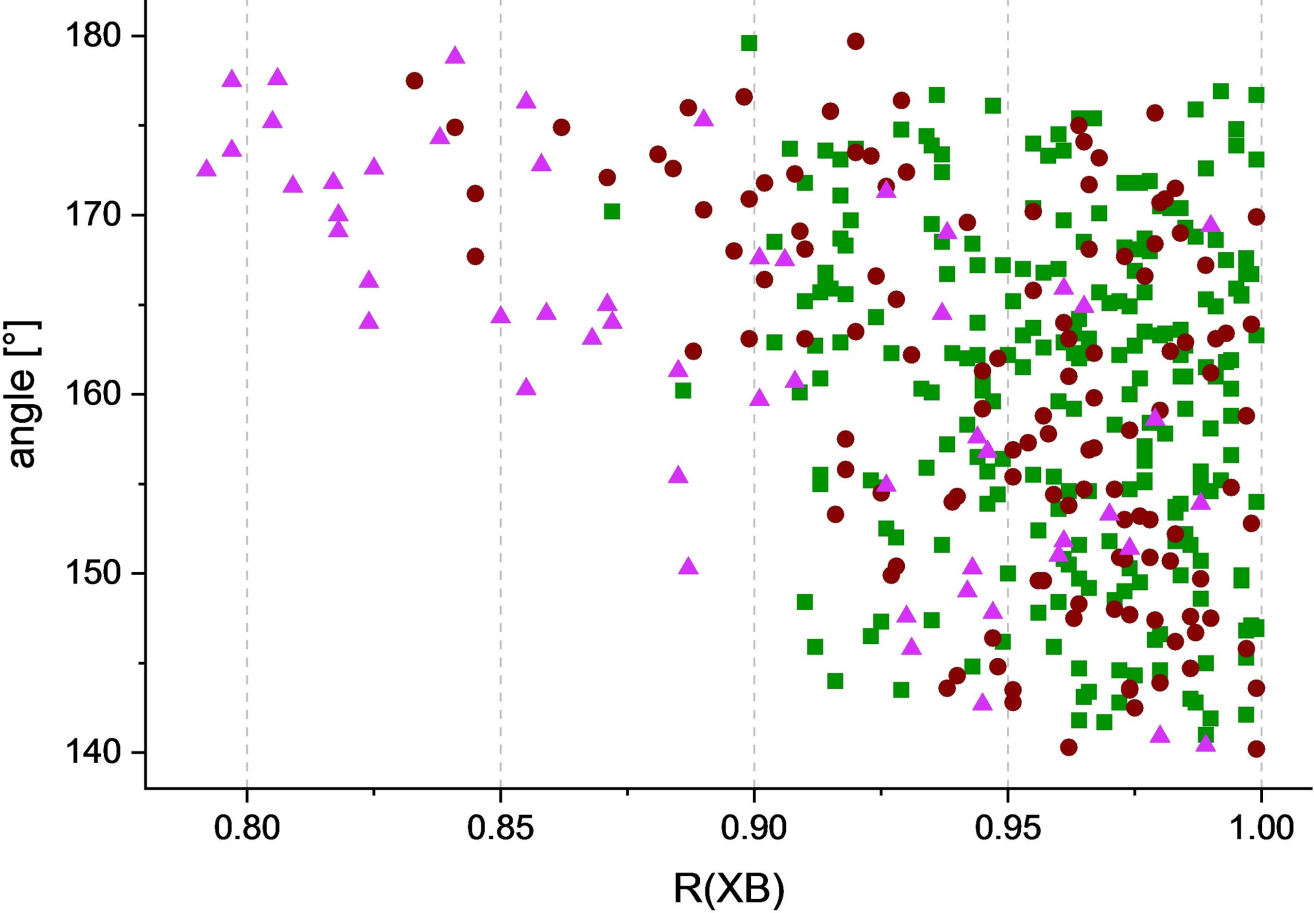
Representation of the bonding parameters (R_XB_ values and ∡_R‐X⋅⋅⋅Y_‐angles) found in the crystal structures of halogenated anions found in the CSD (pink triangles: X=I, brown dots: X=Br, green squares: X=Cl).

Analyses of crystallographic databases demonstrate that several examples of “anti‐electrostatic” halogen bonding were observed experimentally in the solid state. In fact, a survey of the crystal structure database performed by Shi, Zhu and co‐workers in 2016 (as a part of their computational analysis of AEXBs) yielded 119 anion‐anion XB interactions in 99 crystal structures (79 structures for X=Cl, 29 for X=Br and only 11 for X=11).^[21]^ Their study, however, was limited to oxygen‐based Lewis bases. Thus, a more general database survey (CSD version: 5.41) was performed in the context of this concept article using the following criteria: i) the R−X⋅⋅⋅Y (X=I, Br, Cl) distance is shorter than the sum of the respective van der Waals radii, ii) the interaction angle ranges between 140 and 180° and iii) the species forming the halogen bonded complex are both anionic. This search revealed 409 contacts in crystal structures of 32 iodinated, 87 brominated and 176 chlorinated organic or organometallic anions.^[29]^ The geometric characteristics of these contacts (bond angle vs. the ratio of the interatomic distances to the sum of the van der Waals radii, R_XB_), are summarized in Figure 6.

Besides the systems included in Figure 6, a large number of structures containing chlorinated molecules were found which were not further analysed, since these species generally showed very weak interactions, with only three structures exhibiting R_XB_‐values^[30]^ below 0.90 (2 % of all R−Cl⋅⋅⋅Y contacts found). In contrast, nine such crystal structures with X=Br (10 %) and 18 (56 %) involving iodinated anions were found. Figure 6 clearly shows that the iodinated species form the shortest and the most linear halogen bonds. The associations of brominated XB donors are found mostly in the medium region and chlorinated anions are predominantly involved in very weak interactions. Accordingly, the average R_XB_‐values of the analysed structures are 0.895 (X=I), 0.950 (X=Br) and 0.961 (X=Cl), respectively. This data confirms the results of the theoretical studies which predict that the interaction strength of AEXBs (as well as of common XBs) increases in the row Cl<Br<I.

**Figure 7:**
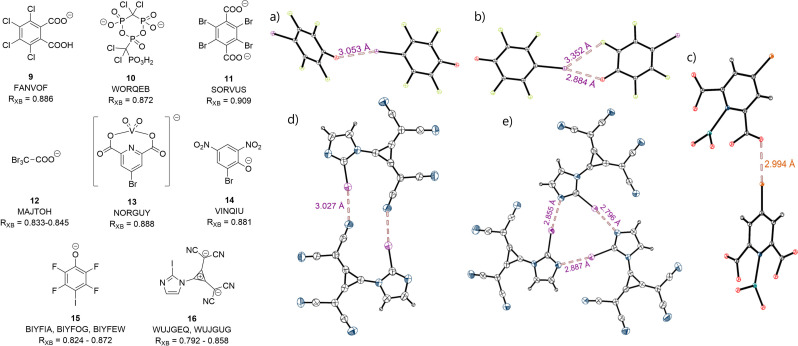
Selected Lewis structures (left) and crystal structures (right) of unimolecular XB complexes with short interaction distances, including CSD reference codes. All counterions and solvent molecules were omitted for clarity. Thermal ellipsoids at 50 % probability. a) BIYFEW, b) BIYFOG, c) NORGUY, d) WUJGEQ, e) WUJGUG. (black: carbon, blue: nitrogen, purple: iodine, red: oxygen, orange: bromine, green: fluorine, teal: vanadium).

Most of the XB donors found in these structures are benzene derivatives, whereas non‐aromatic compounds are usually either based on 1,4‐benzoquinone scaffolds or on halogenated acetate derivatives. In almost all cases, the overall anionic charges of the XB donors are related to the presence of sulfonate (R−SO_3_^−^), carboxylate (R−COO^−^) or R−O^−^ substituents. In addition, some of them comprise anionic R−OPO_3_H^−^, R−SO_2_−N−SO_2_−R^−^ or R−C(CN)_2_^−^ functional groups. Consequently, the atoms involved in these XB contacts are commonly halogen or oxygen atoms. Interestingly, the XB contacts involved, in some cases, dianions despite their seemingly even more repulsive charges (see for example, **10** ^[31]^ and **11** ^[32]^ in Figure 7, left; see Supporting Information for more details).

It should be stressed, however, that with a few exceptions (see below), the short contacts illustrated in Figure 6 were observed in crystals comprising only one self‐associated anionic species. Typically, these contacts involved the halogen substituent in one anion and an electron rich center in another identical anion. This led to a variety of structural motifs in these single‐component salts ranging from simple dimers or 1D‐chains to complex 3D‐networks. A selection of representative structures which exhibit contacts at least 9 % shorter than the sum of the respective van der Waals radii is depicted in Figure 7. In the case of compounds **9**,^[33]^ **11**, **13**,^[34]^ **14**,^[35]^ and **15**,^[36]^ the XB donors form infinite chains, the anions of tribromoacetate **12** ^[37]^ form a 2D‐honeycomb network, and the dianions **2** are arranged in dimers. It should be also mentioned that due to the relatively weak interaction between anions, the structural features of their self‐associations can be substantially affected by crystal forces and counterions. Examples of counterion‐induced structural differences in the salts of 2‐iodoimidazolium derivative **16** and 2,3,5,6‐tetrafluoro‐4‐iodophenolate **15** are illustrated in Figure 7. While the distinctions in the self‐associations of anions **15** in the salts with different cations are rather minor (Figure 7a and b), the structures comprising anions **16** and different counterions result in either symmetric dimers or a trimeric motif (Figure 7d and e).^[13]^ Note that the interaction distances in the trimer of **16** are the shortest XBs that were found in the context of this database survey.

**Figure 8:**
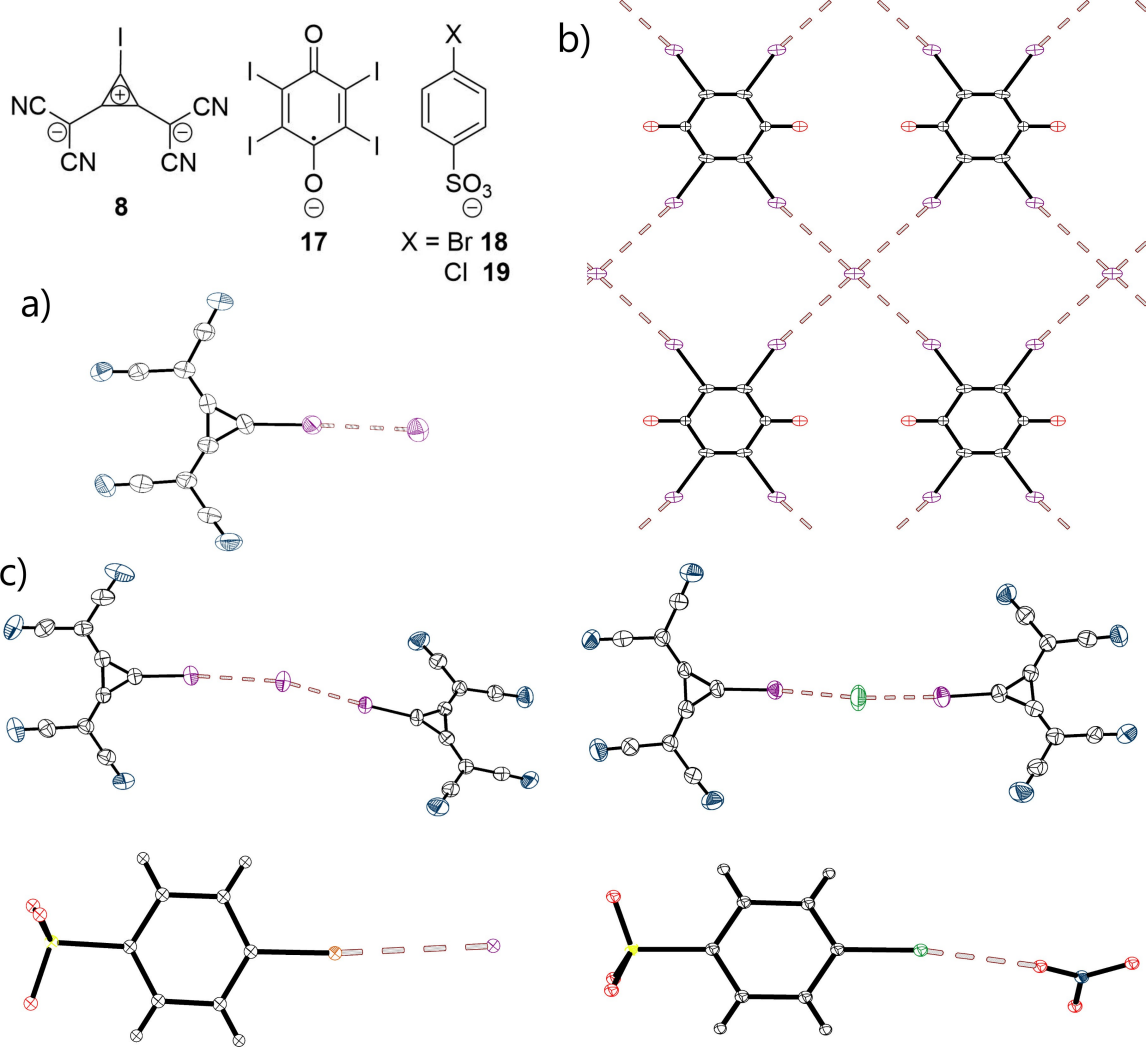
X‐ray structures of the co‐crystals showing AEXBs between a) radicals of tetraiodo‐*p*‐benzoquinone **17** and iodide anions, 1,2‐bis(dicyanomethylene)‐3‐iodo‐cyclopropanide **8** and iodide (b+c) or chloride (d), 4‐bromobenzenesulfonate **18** and iodide (e) and f) 4*‐*chlorobenzenesulfonate **19** and nitrate. All counterions and solvent molecules, as well as the disorder of the chlorobenzene‐ring, are omitted for clarity. The chloride ion in the 1 : 2 co‐crystal (**8**_2_⋅Cl^−^) (d) is disordered and replaced with iodide (Cl : I=8 : 2) (black: carbon, red: oxygen, purple: iodine, green: chlorine, blue: nitrogen, yellow: sulphur, orange: bromine).

While AEXB leading to self‐associations of anionic species represent an important factor in determining their structural motif, it does not affect the composition of the (single component) crystals described above. It is thus important to identify halogen bonding which brings together distinct anions and thus leads to the formation of co‐crystals which would not exist without this “anti‐electrostatic” interaction. Out of all 295 crystal structures analysed, only six co‐crystals were found which show interactions between anionic XB donors and structurally different anions (halides or nitrate). All these co‐crystals are depicted in Figure 8. Their geometrical characteristics indicate that the chlorinated and brominated XB‐donors form rather weak halogen bonds with another anion, as the XB distances in these co‐crystals are merely 4–7 % shorter than the sum of the vdW radii (for I^−^ and Cl^−^, the corresponding Pauli radii for ions^[38]^ were applied). Additionally, the bonding angle for the complex involving 4‐chlorobenzenesulfonate **19** differs significantly from the ideal angle of 180°.

Recently we reported that 1,2‐bis(dicyanomethylene)‐3‐iodo‐cyclopropanide **8** forms halogen bonds in the solid state despite its negative charge.^[13]^ The AEXB with participation of this compound led to formation of unimolecular, self‐assembled chains with R_XB_=0.84. Moreover, this anion was co‐crystallized with chloride and iodide salts. These co‐crystals show either a 1 : 1 or 1 : 2 interaction (see Figure 8a and c‐d). In all three complexes, the XB contacts are significantly shorter than the sum of the vdW radii with R_XB_=0.797‐0.817. Consequently, these contacts rank among the shortest AEXBs analysed in here (see also Figure 6).

**Figure 9:**
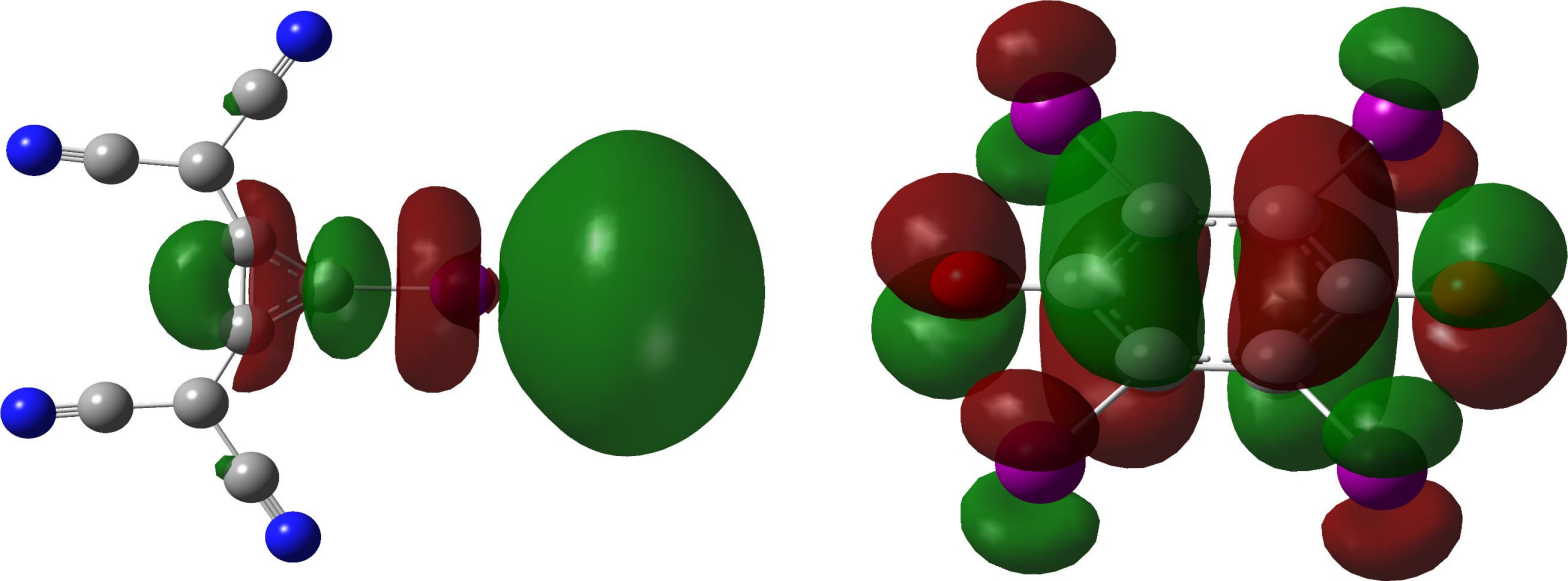
Calculated LUMO of 1,2‐bis(dicyanomethylene)‐3‐iodo‐cyclopropanide **8** (lef) and SOMO of the tetraiodo‐*p*‐benzoquinone radical **17** (right) calculated on the M06‐2X/def2‐TZVP level with additional diffuse function for iodine.

The anionic radicals of tetraiodo‐*p*‐benzoquinone **17** provide another example (next to the three structures involving **8**) of solid‐state AEXB with different anions, here iodide.^[15]^ As depicted in Figure 8b, halogen bonding between these distinct anions led to the formation of 2D‐layers. The XB‐distances are significantly longer than for the previously discussed co‐crystals of **8** (see Table 2). These differences might be attributed to a diverging nature of bonding in these systems. Indeed, the electrostatic potentials calculations revealed that polarization by the nearby halide anions creates positively charged σ‐holes on the surfaces of the iodine substituents in XB donors **8** and **17**, which contribute to attractive interactions in both systems. However, the molecular‐orbital interactions between halides with these XB are quite different. As illustrated in Figure 9, the lowest unoccupied σ*‐orbital of (LUMO) of **8** expands predominantly along the extension of the C−I‐bond. This facilitates its interaction with the HOMO of halide anion (and enables n→σ* charge transfer). In comparison, a frontier orbital of **17** relevant for the MO interaction with the halide is a single occupied molecular orbital (SOMO) delocalised over the whole radical anion moiety. The presence of an electron and delocalization hinder the bonding interaction of the SOMO with the HOMO of the halide (and intermolecular charge‐transfer). Hence, while charge‐transfer might play a significant role in XBs involving **8**, the electrostatic component and polarisation are dominant factors in the XB of **17** (note, however, that this suggestion is based on the consideration of just two available systems and further analysis of appropriate examples are required for its verification.)

**Table 2:** Geometrical parameters for six co‐crystals exhibiting XB contacts between two or more anions.

Ref.code		X	LB	d_XB_	R_XB_	∡_R‐X⋅⋅⋅LB_
WUJGAM^[13]^	**8**	I	I^−^	3.331	0.797	177.5
WUJGIU^[13]^	**8**	I	Cl^−^	3.022	0.797	173.6
		I	Cl^−^	3.051	0.805	175.2
WUJGOA^[13]^	**8**	I	I^−^	3.337	0.806	177.6
		I	I^−^	3.416	0.817	171.8
FUWFOV^[15]^	**17**	I	I^−^	3.920	0.938	169.0
TUPKIZ^[39]^	**18**	Br	I^−^	3.637	0.930	172.4
DISJOE^[40]^	**19**	Cl	NO_3_^−^	3.137	0.959	145.9

**Figure 10:**
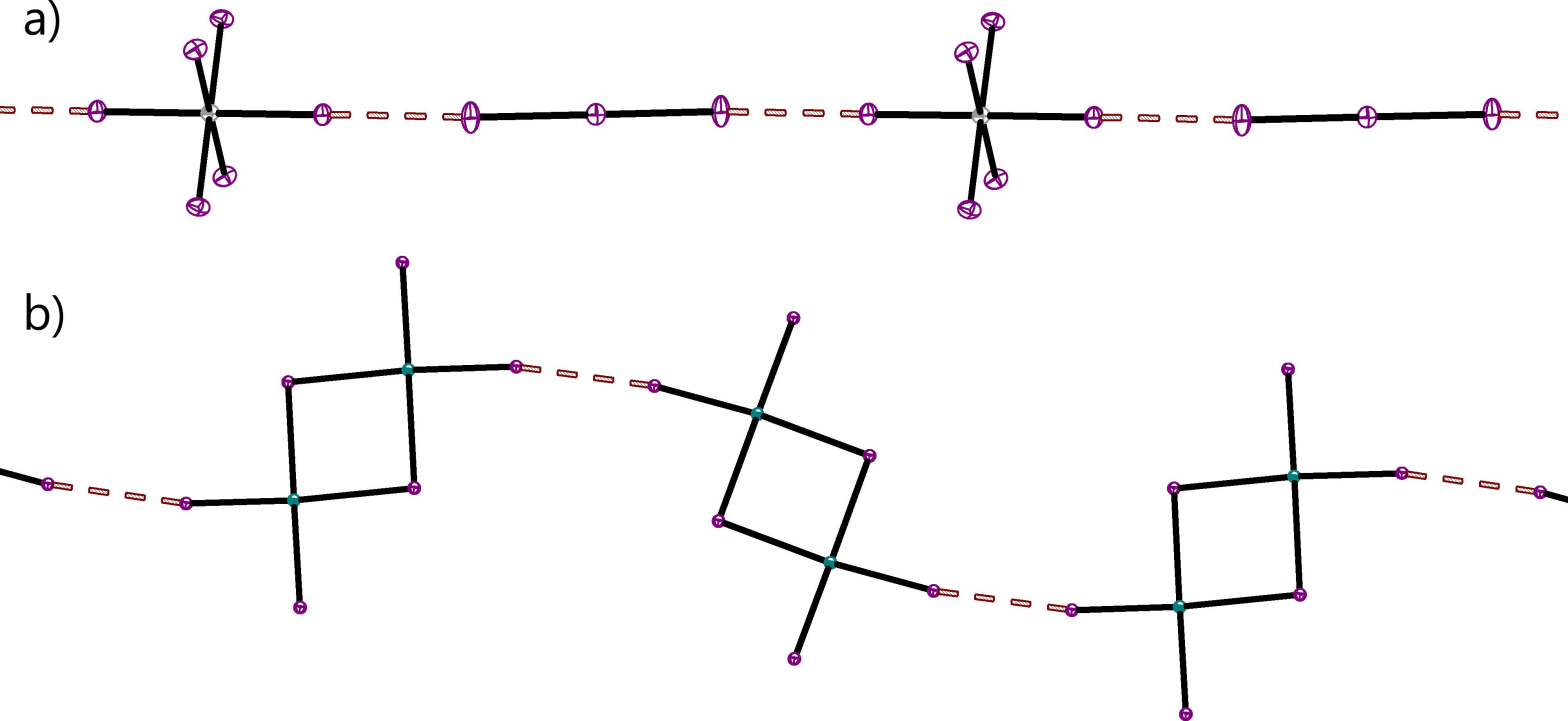
a) Infinite halogen bonded chains in the co‐crystal between bis(dimethylammonium) hexaiodoplatinum(IV) and dimethylammonium triiodide (CSD‐number: 1864903).^[41]^ b) Self‐assembled chains of bis(triethylhydroxyphosphonium) bis(triethylphosphine oxide) bis(m_2_‐iodo)‐tetraiodo‐di‐tellurium (Ref.‐Code: CEMMEN)^[42]^ (counterions omitted for clarity, purple: iodine, teal: tellurium, grey: platinum).

While the focus of the CSD analysis was on organic and organometallic XB donors, we also found various examples of AEXBs involving inorganic compounds. The XB contacts in these structures typically show R_XB_‐values above 0.95. A remarkable exception is found for the interaction between anionic hexaiodoplatinum(IV) and triiodide. In this crystal structure, I_3_^−^ bridges the PtI_6_^2−^ units, thus forming infinite chains with R_XB_=0.88 and ∡_Pt‐I‐I_=179° (see Figure 10a).^[41]^ Another example is depicted in Figure 10b, showing the self‐assembled chain‐motif of bis(m_2_‐iodo)‐tetraiodo‐di‐tellurium (R_XB_=0.92, ∡_Te‐I‐I_=170°).^[42]^

Polyhalides represent another class of inorganic compounds showing halogen bonding involving anionic counterparts. A large variety of these species, usually depicted as X_2*m+n*_^*n−*^ (*m* and *n* integers >0, *n*=1‐4, *m* is equal to the number of halogen molecules and *n* is equal to the number of halide ions from which the polyhalide is build, for example *m* I_2_+*n* I^−^) have been structurally characterised.[^43^, ^44^, ^45^] The diversity of these structures is related to the remarkable propensity of iodine to form noncovalent / hypervalent bonds and thus to catenate via donor‐acceptor interactions.^[43]^ The stability of the polyhalides is determined by their general composition (an increase in iodine content leads to a destabilisation of the resulting compounds),^[43]^ as well as by the size, charge, shape, and symmetry of the counterions. The latter have substantial impact on the structural features of the polyhalide salts, which range from the simple 1D‐chains to complex 2D‐ or 3D‐networks.^[43]^

**Figure 11:**
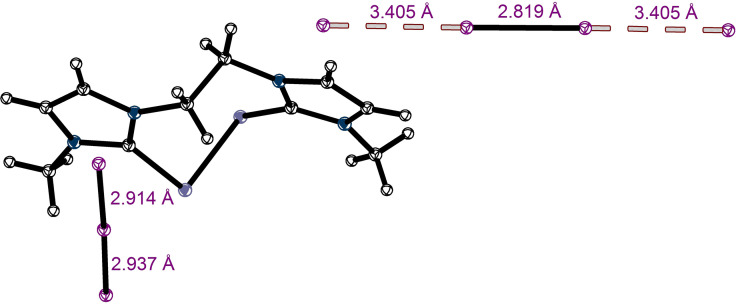
Crystal structure involving both I_3_^−^ and I_4_^2−^ which is of the form [(2I^−^)⋅I_2_]. The relevant bond lengths are given. (Ref.‐code: ALEBAW,^[47]^ purple: iodine, blue‐grey: selenium, blue: nitrogen).

It should be noted, however, that the interaction in the I_*2n+2*_^*2−*^ polyiodides (e. g., I_4_^2−^ with *n*=1) can be interpreted as the interaction of two anions with one central neutral molecule [I^−…^I_2_^…^I^−^] or as the interaction between two anions [I^−…^I_3_^−^]. A theoretical study of bonding within these species by Wang, Shaik, Mo et al. revealed destabilising electrostatic terms, comparably weak polarisation contributions and charge transfer interactions as the most important energy components. This suggested that polyhalides ([Cl(I_2_)_4_]_2_^2−^ and [Cl_2_(I_2_)]^2−[46]^) can be regarded as being “anti‐electrostatic”.^[25]^ Yet, an analysis of the bond distances in the crystal structures of I_4_^2−^ indicated that this dianion can be more realistically described as an [I^−…^I_2_^…^I^−^] association,^[43]^ as shown in the example of a crystal reported by Taouss and Jones (Figure 11).^[47]^ Similar to the other polyhalides, structural features of these associations are significantly affected by counterions, which are also critical for the stabilization of the I_4_^2−^ dianion.^[43]^ The analogous Br_4_^2−^ also exhibits the [(2Br^−^)⋅Br_2_] configuration. For further information on other representatives of the X_*2n+2*_^*2−*^‐group (X=I, Br, Cl) see the comprehensive overview by Svensson and Kloo^[43]^ and reviews by Riedel and co‐workers.[^44^, ^45^]

In summary, the CSD analysis revealed a number of halogen‐bonded associations between ions of like charges although many of these interactions seem to have gone unnoticed. In the vast majority of cases, AEXBs were found in single‐component crystals. However, a very limited number of co‐crystals showing halogen bonds between organic anions and halides or nitrate were also identified. This demonstrates that anionic XB donors might be applied in crystal engineering or medicinal chemistry.^[20]^ The latter is also supported by a study by Valencia et al. showing that the formation of negative halogen bonding can lead to an increase in ligand biological activity.^[48]^

Since arrangements of ionic species in the solid state are affected by the counter‐ions and by crystal forces, the experimental verification of the intrinsic ability of two anions to form a stable XB complex (with potential chemical and biochemical applications) requires the identification of XB associations in the liquid or gas phase. Yet, only the polyhalide species I_4_^2−^ (which could be interpreted as an association of a diiodine XB donor with two iodide XB acceptors, see above) were previously observed in solution.^[49]^ It is important, therefore, that our latest UV‐Vis studies of the interaction between the cyclopropenylium‐based XB donor **8** and halide anions demonstrated spontaneous formation of the AEXB complexes in moderately‐polar and polar solvents.^[14]^ Quantitative treatment of the UV‐Vis spectral changes produced formation constants of 15 M^−1^, 17 M^−1^ and 40 M^−1^ for the AEXB complexes **8**⋅I^−^, **8**⋅Br^−^ and **8**⋅Cl^−^, respectively, in acetonitrile and 8 M^−^1 for the **8**⋅I^−^ complex in dichloromethane. These values correspond to free energy changes of complex formation in a range of −5 to −8 kJ/mol.^[14]^ Notably, they are comparable to those reported for XB complexes of halides with the most common neutral XB donors.[^2^, ^50^] Thus, in moderately polar and polar solvents, which attenuate the electrostatic anion‐anion repulsion and facilitate close approach of the interacting species, halogen bonding between anionic XB donor **8** and halides is sufficiently strong to overcome electrostatic repulsion between the two anions. Further research is needed, however, to find whether other (if any) halogen bonded complexes between ions of like charges can exist beyond the solid state.^[51]^

## 4 Other “Anti‐Electrostatic” Interactions

**Figure 12:**
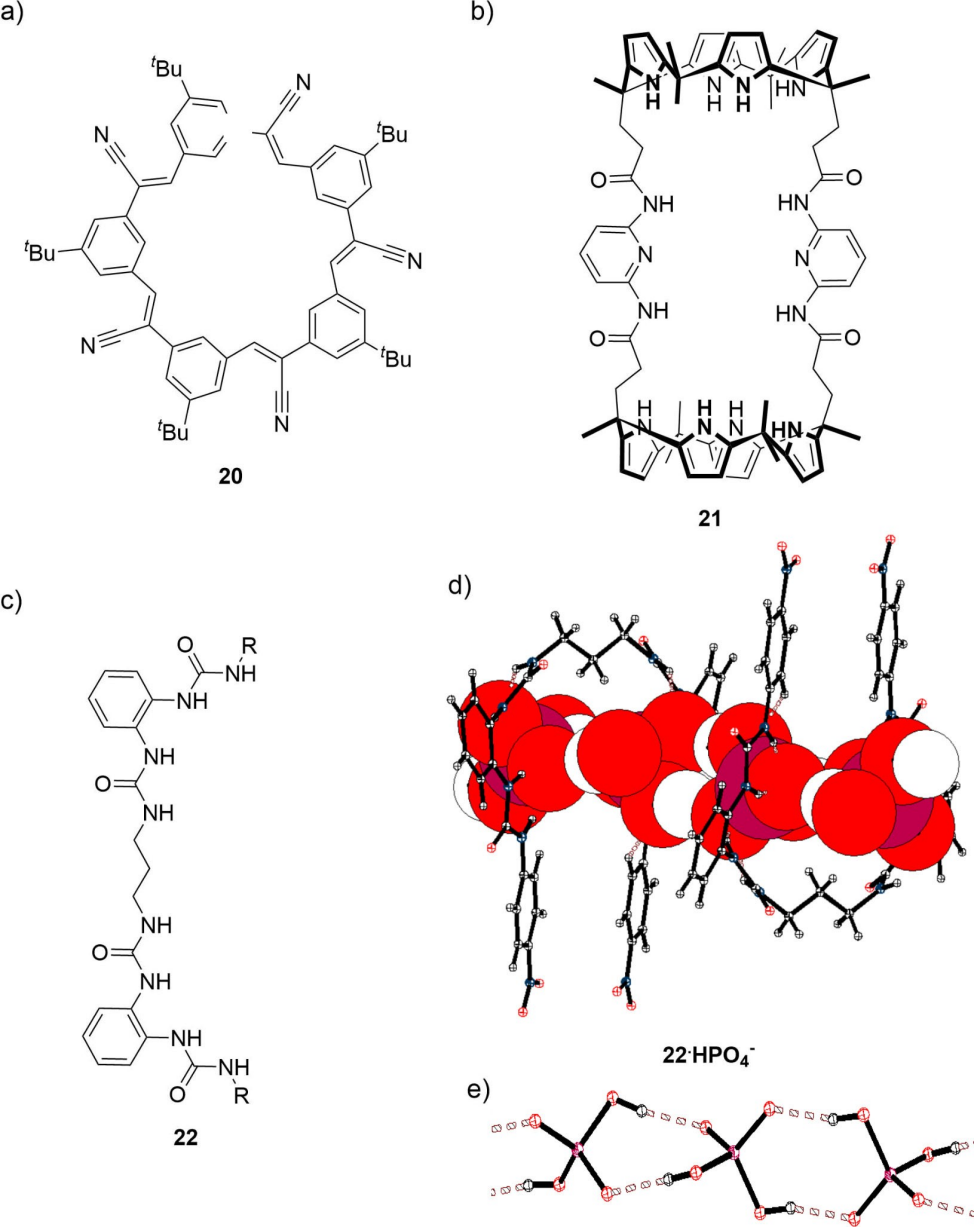
Lewis representations of receptors which were found to stabilise AEHBs in solution: a) The cyanostar macrocycle developed by Flood et al. b) Bis‐calix[4]pyrrole which was used to encapsulate for example sulfate dimers. c) open‐chain receptor which stabilises 1D‐chains of HPO_4_^−^ (d) and e). d) Receptor shown as ellipsoids and (HPO_4_^−^)_n_ in the space‐filling representation. e) Crystal structure of (HPO_4_^−^)_n_. (red: oxygen, white: hydrogen, pink: phosphorous, blue: nitrogen).

Significant attention of the scientific community was focussed recently on “anti‐electrostatic” hydrogen bonds (AEHB). Several theoretical[^17^, ^52^] and experimental studies discussing this concept will be briefly surveyed in this paragraph. A more detailed review was recently published by Flood and White, and we refer interested readers to this source.^[53]^ In the solid state, AEHB complexes predominantly involve OH‐residues of phosphates, sulphates and carbonates. These complexes are usually found in single‐component crystals (with only one example of a co‐crystal) and they show short O−H⋅⋅⋅O contacts in the range of 2.5 to 2.7 Å,^[53]^ corresponding to R_HB_‐values of 0.82–0.89. The interaction distances in the case of carboxylate dimers were found to be similar or even slightly shorter than the respective interaction distances in the dimers of a neutral carboxylic acid. This demonstrates that ‐ at least in the solid state ‐ the interaction between two anionic compounds can be as strong as the more common interaction between neutral binding partners. AEHB‐induced self‐associations of H_2_PO_4_^−^, H_2_PO_7_^2−^, HSO_4_^−^, SO_4_^2−^ or organophosphates in the presence of additional stabilising receptors were also studied in solution. Measurements by NMR‐spectroscopy,[^54^, ^55^, ^56^, ^57^, ^58^, ^59^, ^60^, ^61^, ^62^, ^63^] UV‐vis[^55^, ^56^, ^64^] or ITC titrations[^55^, ^56^, ^57^, ^58^] showed that the AEHB involving these anionic species can result in the formation of well‐defined complexes which are stable in very polar solvents. In fact, the DNA double helix could also be considered as an example here, as the two anionic strands are held together by noncovalent interactions (hydrogen bonds, dispersion, and the hydrophobic effect). Application of neutral open‐chain or macrocyclic receptors (which can interact as a Lewis acid with the anionic species, thus buffering the electrostatic repulsion between the anionic binding partners) made it possible to detect receptor‐stabilised complexes even in the gas phase using ESI‐MS techniques.[^56^, ^58^, ^60^] Examples for receptors which were used in these works are depicted in Figure 12. In particular, the so‐called cyanostar macrocycle **20** designed by Flood et al. stabilized HSO_4_^−^ dimers,[^54^, ^59^] H_2_PO_4_^−^ dimers and trimers^[60]^ or organophosphate dimers.^[61]^ Sessler and co‐workers used the bis‐calix[4]pyrrole **21** to encapsulate dimers of sulfate and H_2_P_2_O_7_^3−^ (Figure 12 b^[55]^ and Figure 12d). Figure 12e depicts the crystal structure of a dihydrogen phosphate polymer which is accomplished by an open‐chain alkanediyl‐spaced bis(bisurea) receptor **22** (see Figure 12c).^[65]^ Anion‐anion HBs were also applied in other areas of supramolecular chemistry and enabled the formation of supramolecular polymers[^63^, ^66^] and hydrogen bonded organic frameworks in the solid state.^[67]^

**Figure 13:**
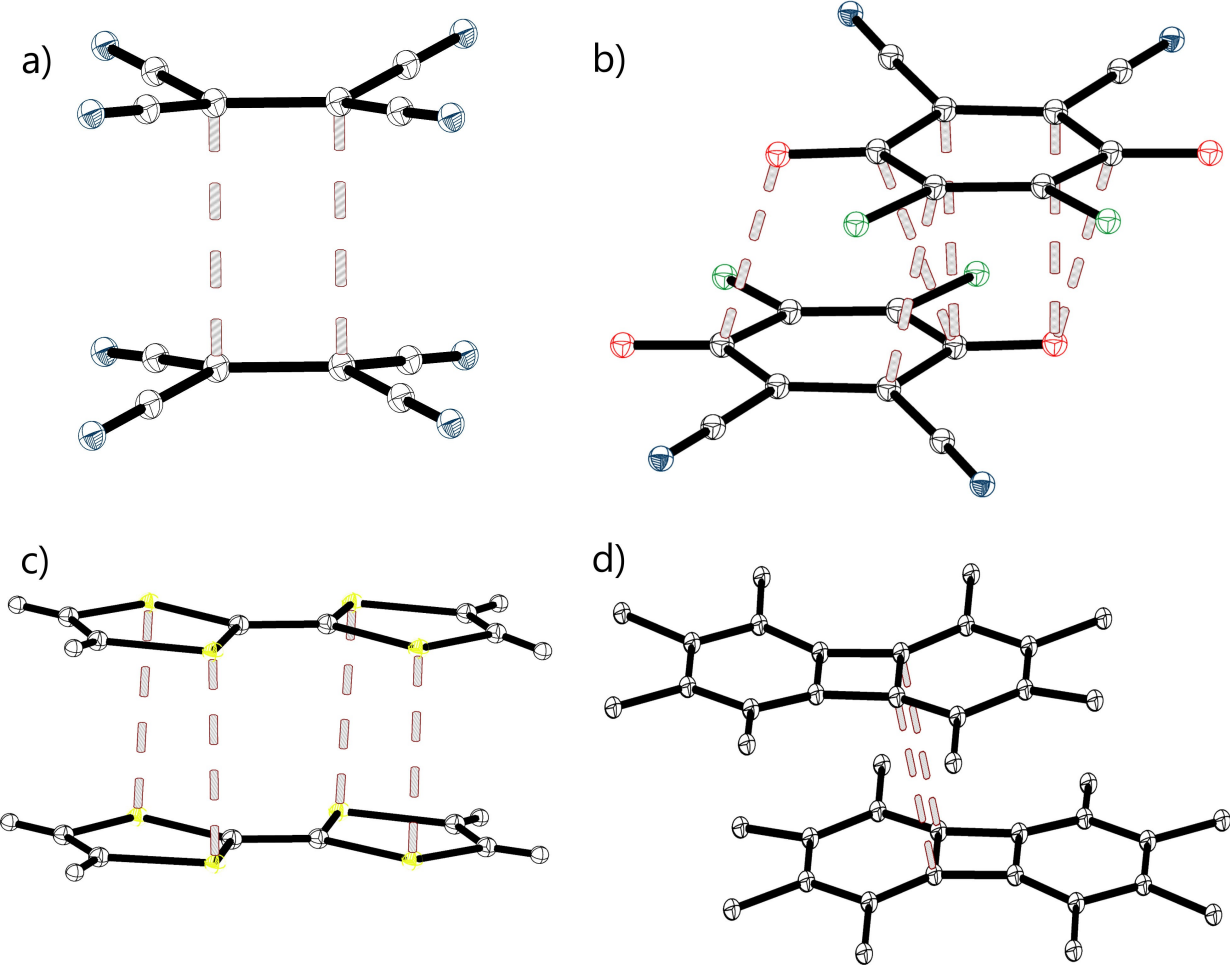
Crystal structures of the π‐bonded (dianionic or dicationic) dimers formed by anion and cation radicals: a) (TCNE^−^)_2_ (Ref.‐Code: ODAFAA),^[73]^ b) (DDQ^−^)_2_ (Ref.‐Code: DOVWIV),^[75]^ c) (TTF^+^)_2_ (Ref.‐Code: ZZZBWA10)^[76]^ and d) (OMB^+^)_2_ (hydrogen atoms of methyl groups omitted for clarity; Ref.‐Code: QETFIE).^[77]^

Multicentre bonding between two radical anions or cations (commonly referred to as pancake bonding) leading to the formation of dianionic or dicationic π‐dimers represents another example of an intermolecular “anti‐electrostatic” interaction.[^68^, ^69^, ^70^] Such discrete dimeric units consisting of π‐stacked radical‐ion moieties were identified in many salts of planar π‐conjugated radical ions.^[71]^ For example, tetracyanoethylene (TCNE) radical anions form π‐dimers (TCNE)^2‐^ in which eclipsed TCNE moieties are arranged atop each other (Figure 13a).[^72^, ^73^, ^74^] The intra‐dimer C⋅⋅⋅C distances vary between 2.8 Å and 3.0 Å in the salt with different counterions (i. e. R_CC_∼0.85), which suggests strong attraction between the anionic counter‐parts.

The dianionic π‐dimers of dichlorodicyano‐*p*‐benzoquinone (DDQ) comprised pairs of the DDQ moieties slipped relative to another along or perpendicular to the main O−O axes (Figure 13b).[^74^, ^75^, ^78^] They are characterized by an interplanar separation of about 2.9 Å and show multiple short C⋅⋅⋅C and C⋅⋅⋅O contacts in the 2.9–3.0 Å range. Similar dianionic π‐dimers with eclipsed, slipped or rotated counter‐parts were identified in the salts of other anionic *p*‐benzoquinone derivatives, tetracyanoquinodimethane, etc.[^69^, ^70^, ^74^, ^75^, ^78^, ^79^, ^80^, ^81^, ^82^] Many π‐conjugated radical cations also show a high tendency to form dicationic π‐dimers. Such “anti‐electrostatic” associations were identified in a large number of salts of oxidized tetrathiafulvalene, TTF (Figure 13c) and its analogues, octamethylbiphenylene (Figure 13d).[^69^, ^70^, ^76^, ^83^, ^84^, ^85^, ^86^] Solid‐state electron paramagnetic resonance (EPR) studies revealed the diamagnetic nature of these π‐dimers which show spin‐paired (singlet) ground states.[^74^, ^75^, ^79^, ^80^, ^83^] Solid‐state UV‐Vis measurements showed the appearance of new absorption bands of the dimers in the NIR range and Davydov's blue shift of the intra‐monomer transitions in the dimerized species.[^74^, ^85^]

Variable‐temperature UV‐vis and EPR measurements of the solutions of the aforementioned ion‐radicals salts demonstrated that these cationic or anionic species (e. g. TCNE^−.^, DDQ^−.^, TTF^+.^, etc.) form π‐dimers in polar solvents.[^79^, ^83^, ^85^] Thermodynamic characteristics of these associations resulting from above‐mentioned measurements revealed that the intrinsic stability of the π‐dimers is derived from the relatively high magnitudes of the negative enthalpy changes (ΔH) of dimerization (mostly in the −20 to −50 kJ/mol range).[^74^, ^86^] At high temperatures, they are compensated by the negative values of the entropy changes, ΔS (between −100 to −200 J mol^−1^ K^−1^). As such, the formation constants (and concentration) of π‐dimers at room temperature are very small, but they increase considerably at lower temperatures.

Computational studies showed that similarly to AEXB and other supramolecular interactions, dispersion, counter‐ions and/or polarity of the media are important factors in such “anti‐electrostatic” bonding between radical ions in the solid state and in solution. Yet, the stability of the π‐dimer and their structural and spectral features could be accounted only if molecular‐orbital interactions are taken into account.[^68^, ^74^, ^79^, ^80^, ^81^, ^82^] This weakly‐covalent contribution is related to the formation of bonding orbitals of the dimers from the semi‐occupied orbitals (SOMO) of radical ions. In the halogen‐bonded complexes, the analogous bonding orbitals are derived from the HOMO of the electron donor (XB acceptor) and the LUMO of the electron acceptor (XB donor), and their formation is accompanied by a partial charge transfer between interacting species. The ongoing discussion on the role of such charge‐transfer interactions in XB vs. a solely electrostatic model (including dispersion) in which the shift of the electron density in XB associations is described as a polarization of their counter‐parts, necessitates a clarification of the distinctions and implications of these concepts.

## 5 Comments: Charge‐Transfer vs. Polarization‐Only a Semantic Difference?

Conceptually, the distinction between polarization and charge transfer seems clear – the former represents a shift of the electron density within individual components of the complex, while the latter describes an electron density movement from one of these species to another.^[87]^ One may argue, however, that these processes are physically indistinguishable, and therefore there is no need to invoke charge transfer. In other words, electrostatics may provide a complete picture of halogen bonding if polarization is taken into account.^[88]^

It should be mentioned in this respect that examples of electron density movement *between* interacting species can be found in the studies on π‐bonded complexes formed by ion‐radicals with their parent closed‐shell molecules. The doubling of the hyperfine splitting of the EPR signals (and halving of the hyperfine splitting constant) of such associations as compared to the individual ion‐radicals showed that the unpaired electron is delocalized over the whole complex (i. e., electron density is transferred from one moiety to another).^[89]^ In the case of the XB complexes, the charge transfer between interacting species (as opposed to their polarization) was identified experimentally using X‐ray absorption spectroscopy in works of Kennepohl et al.^[90]^ The appearance of new peaks in the K‐edge X‐ray absorption spectra of chloride indicates that a physically measurable amount of charge was transferred from this anion to the XB donors in strong and moderately‐strong XB complexes.

The population of the antibonding orbital of the XB donors as a result of a partial charge transfer weakens the C−X bond and facilitates thermal and photochemical reactions with participation of the halogenated species.[^91^, ^92^] While polarization implies that partially deformed reactants remain essentially isolated species, charge transfer leads to a joint entity with a common molecular orbital system showing diagnostic UV‐Vis bands.[^10^, ^93^] Their irradiation results in complete electron transfer which could be fruitfully utilized in synthesis.[^91^, ^92^, ^94^] The intensity of the absorption band is determined by the magnitude of the electronic coupling of the reactants. The latter attenuate the barrier for the thermal electron transfer (and follow‐up reactions) and the rate of such processes can be predicted from the spectral and structural characteristics of the complex using Marcus‐Hush theory.^[95]^ Finally, experimental measurements of the charge density provided another example of a clear distinction between charge‐transfer and polarization and pointed out a partial covalent nature of halogen bonding.^[9]^ A progressive increase of the contribution of the charge‐transfer (weakly‐covalent) component allows to rationalize the continuum of XB lengths and energies, ranging in many series of XB complexes without substantial gap from weak supramolecular association to that of a fully developed covalent bond.^[96]^

Overall, these studies demonstrate that besides the theoretical analyses of XB components (which are based on the subjective choice of computational method), polarization and charge‐transfer can also be differentiated experimentally by physical methods. Most importantly, the concept of charge‐transfer (weakly‐covalent) interactions allows to rationalize‐and to predict‐vital characteristics of many XB complexes and of reactions facilitated by this interaction.

## 6 Summary

The electronic nature and origin of halogen bonding is still under quite intense discussion.^[97]^ An important electronic component is undoubtedly the electrostatic interaction between neutral or cationic halogen bond donors and Lewis bases. Sometimes, halogen bonding is seen as a purely or predominantly electrostatics‐based interaction (including polarization effects), and in various publications a correlation of the halogen bonding strength of a molecule with its most positive surface electrostatic potential (V_S,max_) is assumed. This reasoning would render anionic halogenated molecules mostly unsuitable to act as halogen bond donors and particularly for halogenated molecules with a negative V_S,max_, a positive binding energy would be expected. However, several computational studies predicted that halogen bonds between ions of like charges can be stable or metastable depending on the respective environment. Crystal structure database analysis confirm that such contacts also exist in the solid state, and two prominent examples[^13^, ^14^, ^15^] of such “anti‐electrostatic” halogen bonds have recently been published.

The collected results presented herein demonstrate that anionic XB donors might become an important motif for crystal engineering. The main tool to influence the compounds’ crystallisation behaviour could be the variation of the counterion as well as the usage of receptors, which interact with the anions. Such receptors were shown to be efficient to stabilise AEHBs both in the solid state and in solution.[^53^, ^98^] Analogously, their usage could also be an efficient tool to stabilise AEXBs. A first experimental study has now demonstrated that AEXBs can be stable enough to be detected in solution.^[14]^ Whether halogen bonded complexes between ions of like charges might find application in solution and if they can be detected even in the gas phase are important questions to be addressed in the future.

## Conflict of interest

The authors declare no conflict of interest.

## Supporting information

As a service to our authors and readers, this journal provides supporting information supplied by the authors. Such materials are peer reviewed and may be re‐organized for online delivery, but are not copy‐edited or typeset. Technical support issues arising from supporting information (other than missing files) should be addressed to the authors.

Supporting Information
