# Supplementary material for: “Anti‐electrostatic” Halogen Bonding between Ions of Like Charge
Source: Chemistry. 2021 Oct 1;27(67):16530–42. doi: 10.1002/chem.202102549 (PMC9293363; doi:10.1002/chem.202102549)
Supplement: Supplementary file 1 — Supporting Information [file CHEM-27-16530-s001.pdf]

Chemistry–A European Journal

Supporting Information

“Anti-electrostatic” Halogen Bonding between Ions of Like Charge

Jana M. Holthoff, Robert Weiss, Sergiy V. Rosokha,* and Stefan M. Huber*

The database survey (CSD version: 5.41) was performed in the context of this concept article using the following criteria: i) the R-X···Y (X = I, Br, Cl) distance is shorter than the sum of the respective van der Waals radii, ii) the interaction angle ranges between 140 and 180° and iii) the species forming the halogen bonded complex are both anionic.

Reference codes of the database, involved atoms on both donor and acceptor side, interaction distances, angles and R_{XB} with $R_{XB} = d_{X\cdots LB}$/(sum of the van der Walls radii of the involved atoms) (in case of halides the Pauli radii were employed), references, as well as additional comments are given in the tables below.

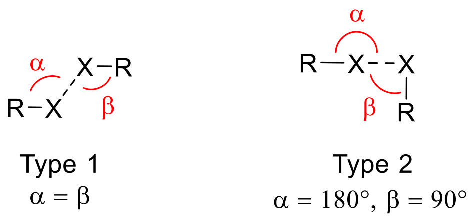

Figure 1. Type 1 and Type 2 halogen bonding in self-complementary XB complexes.

Organic and Metal-Organic Compounds

#	Ref.code	X	LB	$d_{X\cdots LB}$	R_{XB}	∡	∡	Reference	Comment
1	ABUHUA	Cl	Cl	3.402	0.972	144.6	144.6	[1]	Type 1
2	ACITEN	Cl	Cl	3.379	0.965	162.3	162.3	[2]	Type 1
3	AFEDOE	Cl	O	3.198	0.978	171.9		[3]	
4	AGONIV	Cl	Cl	3.478	0.993	167.5	97.9	[4]	Additional interaction with neutral LB
5	AGOTAT	Cl	Cl	3.496	0.999	163.3	163.3	[5]	Type 1
6	AJADAS	Cl	Cl	3.192	0.912	162.7	158.2	[6]	Dianion! Type 1
7	AKIZAV	Cl	Cl	3.3025	0.944	164.0	164.0	[7]	Type 1
8	ALOQIC	Cl	O	3.179	0.972	165.2		[8]	
9	ALOQOI	Cl	Cl	3.4187	0.977	157.1	157.1	[8]	Type 1
10	AMHCLA	Cl	O	3.177	0.972	162.2		[9]	
11	ASIHIV	Cl	Cl	3.406	0.973	149.0	140.5	[10],[11]	Type 1
12	AVOJEB	Cl	Cl	3.363	0.961	173.6	94.6	[12]	dianion
13	BANPUC	Cl	O	3.250	0.994	156.6		[13]	
14	BARLEM	Cl	Cl	3.372	0.963	162.3	156.4	[14]	Type 1
15	BARLIQ	Cl	Cl	3.462	0.989	145.0	145.0	[14]	Dianion! Type 1
16	BEBJIC	Cl	Cl	3.280	0.937	151.6	151.6	[15]	Dianion! Type 1
17	BEKBUP	Cl	Cl	3.199	0.914	166.4	166.4	[16]	Dianion! Type 1, Anionic functionality not in direct conjugation
18	BENBUT	Cl	Br	3.518	0.977	168.7		[17]	
19	BIKCUU	Cl	Cl	3.411	0.974	164.9	129.5	[18]	
		Cl	Cl	3.365	0.961	169.7	82.7		
20	BIKDAB	Cl	Cl	3.431	0.980	146.6	75.0	[18]	
21	BIKVIA10	Cl	Cl	3.274	0.935	160.1	157.7	[19]	Type 1

22	BIRFEP	Cl	N	3.296	0.999	176.7		[20]	
23	BOFBED	Cl	Cl	3.456	0.987	142.8	92.2	[21]	Organometallic species
		Cl	Cl	3.423	0.978	168.0	97.2		
24	BOQHUK01	Cl	O	3.123	0.955	155.5		[22]	
		Cl	O	3.233	0.989	161.5			
25	CANVIW	Cl	Cl	3.181	0.909	160.1	160.1	[23]	Type 1
26	CASHUZ	Cl	O	3.259	0.997	166.7		[24]	Anionic functionality not in direct conjugation
27	CECRAG	Cl	O	3.0895	0.945	160.7		[25]	Additional interaction with neutral LB
28	CECREK	Cl	Cl	3.471	0.992	176.9	82.5	[25]	
29	CEFZUJ	Cl	O	3.021	0.923	155.2		[26]	Anionic functionality not in direct conjugation
		Cl	Cl	3.459	0.988	155.7	121.8		
30	CEKYEW	Cl	Cl	3.3696	0.963	163.4	115.2	[27]	
31	CELHUX	Cl	O	3.222	0.985	169.3		[28]	
32	CEMFIM	Cl	Cl	3.196	0.913	155.0	155.0	[29]	Type 1
		Cl	Cl	3.267	0.933	160.3	160.3		Type 1
33	CEMFIM01	Cl	O	3.121	0.955	163.7		[29]	Additional interaction with neutral LB
34	CENXAX	Cl	Cl	3.414	0.975	144.3	144.3	[30]	Type 1
35	CEPREU	Cl	Cl	3.363	0.961	150.8	150.3	[31]	Type 1
36	CEZSIM	Cl	F	2.939	0.913	166-9		[32]	Organometallic species
37	CIPSUQ	Cl	Cl	3.301	0.943	144.8	144.8	[33]	Type 1
38	CIRTEC	Cl	O	3.062	0.936	176.7		[34]	
39	COFBOO10	Cl	Cl	3.432	0.981	163.4	163.4	[35]	Type 1
40	CUBLAO	Cl	Cl	3.419	0.977	165.7	80.8	[36]	Organometallic species
		Cl	Cl	3.352	0.958	173.3	138.9		
41	CULJOI10	Cl	Cl	3.351	0.957	162.6	122.5	[37]	
		Cl	Cl	3.347	0.956	152.4	124.4		
42	CUKNON	Cl	Cl	3.393	0.969	141.7	141.7	[38]	Type 1
43	CURLIM	Cl	Cl	3.211	0.917	173.1	129.7	[39]	
44	DAQYIG	Cl	O	3.058	0.935	173.9		[40]	
		Cl	O	3.001	0.920	173.7			
		Cl	O	3.222	0.985	152.2			
45	DENQAO	Cl	Cl	3.407	0.973	171.8	171.8	[41]	Type 1, organometallic species
46	DIRFER	Cl	O	3.1366	0.959	145.9		[42]	Co-Crystal
47	DISJOE	Cl	Cl	3.282	0.937	173.4	173.4	[43]	Type 1
48	DITJIA	Cl	Cl	3.452	0.986	151.6	142.2	[44]	Type 1, additional interaction with neutral LB
49	DOJKIY	Cl	O	3.095	0.946	155.7		[45]	
		Cl	O	3.214	0.983	153.7			

50	DOMQOL	Cl	Cl	3.497	0.999	173.1	85.7	[46]	
51	DORBER	Cl	Cl	3.417	0.976	149.5	147.2	[47]	Type 1, organometallic species
52	DOVPIN	Cl	Cl	3.396	0.970	165.1	163.2	[48]	Type 1, organometallic species
53	DOCCIU	Cl	Cl	3.361	0.96	159.6	120.3	[49]	
54	DUBWUS	Cl	Cl	3.278	0.937	168.5	91.2	[50]	
55	DUJCER	Cl	Cl	3.446	0.985	162.7	161.3	[51]	Type 1
56	EBEQAG	Cl	Cl	3.366	0.962	154.6	125.2	[52]	
57	EGABEV	Cl	O	3.166	0.968	165.7		[53]	
58	ERODUL	Cl	Cl	3.425	0.979	146.3	79.8	[54]	
59	ETUPOZ	Cl	Cl	3.495	0.998	147.1	147.1	[55]	Type 1
60	FACTIP	Cl	Cl	3.251	0.929	143.5	143.5	[56]	Type 1
61	FANVOF	Cl	Cl	3.147	0.899	179.6	179.6	[57]	Type 1
62	FEHLUC	Cl	Cl	3.372	0.953	163.3	127.3	[58]	
63	FEMTEW	Cl	Cl	3.444	0.984	162.2	115.0	[59],[60]	
64	FEVJOI	Cl	O	3.002	0.918	165.6		[61]	
65	FITSOQ	Cl	O	3.102	0.949	167.2		[62]	
66	FOPYOB	Cl	Cl	3.305	0.944	167.2	164.5	[63]	Type 1, dianion
67	FOQNUX	Cl	Cl	3.471	0.992	155.2	155.2	[64]	Type 1
68	FOVBUO	Cl	Cl	3.336	0.953	167.0	147.1	[65]	
		Cl	O	3.081	0.942	162.0			
69	FOVBUO	Cl	O	3.081	0.942	162.0		[65]	
70	FOVCAV	Cl	O	3.194	0.977	156.3		[65]	Additional interaction with neutral LB
71	FOWWAQ	Cl	O	3.259	0.997	167.6		[66]	
72	FULPOS	Cl	Cl	3.176	0.907	173.7	173.7	[67]	Type 1
73	FOLPUY	Cl	Cl	3.238	0.925	147.3	147.3	[67]	Type 1
74	GAQTEY	Cl	O	3.262	0.997	142.1		[68]	
75	GEPMAR	Cl	O	3.192	0.976	168.1		[69]	
76	GEPNAT	Cl	Cl	3.448	0.985	159.2	108.4	[70]	
77	GEPYAE	Cl	Cl	3.423	0.978	158.4	108.0	[70]	
78	GIPSEF	Cl	F	3.001	0.934	174.4		[71]	
79	GOKVEH	Cl	O	2.988	0.914	173.6		[72]	
		Cl	O	3.054	0.934	155.9			
		Cl	Cl	3.375	0.964	151.6	151.6		Type 1
80	GOMBEP	Cl	Cl	3.468	0.991	161.0	158.2	[73]	Type 1
81	GOPJEC	Cl	Cl	3.329	0.951	165.2	156.2	[74]	Type 1, organometallic species
82	HATWAB	Cl	Cl	3.374	0.964	164.2	163.9	[75]	Type 1
83	HEFVIY	Cl	Cl	3.485	0.996	165.5	108.6	[76]	
84	HEGFEF	Cl	Cl	3.408	0.974	150.3	105.2	[77]	

85	HIHPOE	Cl	O	3.135	0.959	155.4		[78]	
86	HISREG	Cl	O	3.196	0.977	155.2		[79]	
87	HUFVAG	Cl	Cl	3.429	0.980	170.5	90.7	[80]	
88	HOFZIM	Cl	Cl	3.230	0.923	146.5	146.5	[81]	Type 1
		Cl	Cl	3.324	0.950	162.2	162.2		Type 1
89	HOFZOS	Cl	Cl	3.249	0.928	152.0	152.0	[81]	Type 1
90	HOLPEE	Cl	Cl	3.367	0.962	150.5	150.1	[82]	Type 1
91	IGAKUX	Cl	Cl	3.394	0.970	151.8	150.0	[83],[84]	Type 1
92	IMAYIG	Cl	Cl	3.203	0.915	165.9	142.2	[85]	
93	IMAYOM	Cl	Cl	3.194	0.913	165.7	142.5	[85]	
94	IMAYUS	Cl	Cl	3.186	0.910	165.2	140.6	[85]	
95	IVUKIU	Cl	O	3.107	0.950	150.0		[86]	
96	IVUSIC	Cl	O	3.249	0.994	158.8		[86]	
97	IYEBIX	Cl	Cl	3.457	0.988	150.7	99.8	[87]	
		Cl	Cl	3.498	0.999	146.9	97.9		
		Cl	Cl	3.498	0.99	154.6	105.7		
98	JAMXUR	Cl	Cl	3.484	0.995	173.9	169.6	[88]	Type 1
99	JAVWUZ	Cl	Cl	3.478	0.994	160.3	85.1	[89]	Dianion!
100	JAXJIE	Cl	N	3.215	0.974	154.7		[90]	
101	JAXREG	Cl	O	3.148	0.994	161.9		[91]	
102	JCUJUM	Cl	Cl	3.413	0.975	162.7	161.9	[92]	Type 1
103	JEHPAN	Cl	O	3.141	0.960	174.5		[93]	
104	JEPYAF	Cl	Cl	3.402	0.972	142.8	142.8	[94]	Type 1
105	JEYYAQ	Cl	Cl	3.444	0.984	161.0	85.2	[95]	
		Cl	Cl	3.406	0.973	168.2	168.2		Type 1
106	JIZZIB	Cl	Cl	3.466	0.990	141.9	141.9	[96]	Type 1
107	KEMMOH	Cl	Cl	3.461	0.989	141.0	121.1	[97]	
108	KEQPEC	Cl	O	3.006	0.919	169.7		[98]	
		Cl	O	3.229	0.988	148.6			
109	KHDCMT01	Cl	O	3.206	0.980	144.6		[99]	
		Cl	O	3.236	0.990	158.1			
110	KIDGIQ	Cl	Cl	3.207	0.916	144.0	144.0	[100]	
111	KIDPEV	Cl	Cl	3.238	0.925	154.8	154.8	[100]	Type 1
112	KIJWAD01	Cl	O	3.242	0.991	164.9		[101]	
113	KOVKUB	Cl	Cl	3.342	0.955	170.4	169.2	[102]	Type 1, organometallic species
114	KOYWEA	Cl	Cl	3.443	0.984	170.4	159.7	[103]	
115	KUQRUK	Cl	Cl	3.380	0.966	163.1	124.7	[104]	
116	LARXOU	Cl	O	3.070	0.939	162.3		[105]	
		Cl	Cl	3.452	0.986	143.0	132.9		Type 1

		Cl	Cl	3.347	0.956	147.8	100.0		
		Cl	Cl	3.285	0.938	166.7	137.3		
117	LATROO	Cl	Cl	3.3801	0.966	149.2	103.7	[106]	Organometallic species
118	LATSAB	Cl	Cl	3.374	0.964	141.8	132.5	[106]	Type 1, organometallic species
119	LATSEF	Cl	Cl	3.372	0.963	159.2	116.5	[106]	Organometallic species
		Cl	Cl	3.388	0.968	170.1	95.9		
		Cl	Cl	3.359	0.960	167.0	89.8		
		Cl	Cl	3.399	0.971	158.3	88.3		
		Cl	Cl	3.364	0.961	162.9	102.6		
120	LATSIJ	Cl	Cl	3.410	0.974	160.0	115.2	[106]	Organometallic species
121	LATSOP	Cl	Cl	3.336	0.953	161.5	102.5	[106]	
		Cl	Cl	3.314	0.947	176.1	98.6		
122	LECCOL	Cl	O	3.260	0.997	145.3		[107]	
		Cl	Cl	3.199	0.914	166.8	166.8		Type 1
123	LEMRON	Cl	Cl	3.099	0.948	154.4	154.4	[108]	Type 1
124	LOGLAU	Cl	Cl	3.100	0.886	160.2	160.2	[109]	Type 1, dianion
125	LEPQUT	Cl	O	3.207	0.981	157.8		[110]	
126	LITYUJ	Cl	O	3.124	0.955	174.0		[111]	
127	LIVXIY	Cl	O	3.092	0.946	153.9		[112]	
128	LOFCOA	Cl	O	3.246	0.993	161.8		[113]	
129	LONXIV	Cl	O	3.152	0.964	149.7		[114]	
130	LORWEV	Cl	O	3.068	0.938	157.2		[115]	
131	LORWIZ	Cl	O	3.222	0.985	161.0		[115]	
		Cl	O	3.158	0.966	163.0			
132	LORWOF	Cl	O	2.983	0.912	145.9		[115]	
133	LUKCOL	Cl	N	2.9825	0.904	168.5		[116]	
134	MEPTCP	Cl	O	3.240	0.991	168.6		[117]	
135	MEVYER	Cl	O	3.189	0.976	160.9		[118]	
136	MIVCEC	Cl	Cl	3.485	0.996	165.6	106.5	[119]	
137	NEBYAW	Cl	O	3.219	0.984	162.2		[120]	
		Cl	Cl	3.468	0.991	168.6	102.8		
138	NERJEB	Cl	O	3.174	0.971	148.5		[121]	
		Cl	Cl	3.497	0.999	147.0	130.0		Type 1
		Cl	Cl	3.443	0.984	149.9	136.4		Type 1
139	NIJTOP	Cl	Cl	3.240	0.926	152.5	152.5	[122],[123]	Type 1
140	QELCEQ	Cl	O	3.267	0.999	154.0		[124]	
		Cl	O	3.216	0.983	151.8			
		Cl	O	3.160	0.966	154.6			
141	QISTES	Cl	O	2.957	0.904	162.9		[125]	

142	QUQFEN	Cl	O	3.038	0.929	174.77		[126]	
143	QUXCOC	Cl	O	2.977	0.910	171.8		[127]	
144	RUJQET	Cl	O	3.00	0.917	162.9		[128]	
145	SAQNII	Cl	Cl	3.421	0.977	163.5	142.1	[129]	
146	SAXPOY	Cl	O	3.255	0.995	165.9		[130]	
147	SECXIJ	Cl	O	3.114	0.987	175.9		[131]	
148	SEYBED	Cl	Cl	3.440	0.983	153.4	153.4	[132]	
149	SIMZIY	Cl	O	3.131	0.957	166.8		[133]	
150	SOGVOC	Cl	O	3.160	0.966	143.4		[134]	
151	SRACSN	Cl	O	3.235	0.989	165.3		[135]	Sn-complex
		Cl	O	3.259	0.997	146.8			
152	TECRUP	Cl	O	3.057	0.935	169.5		[136]	
153	TERKOT	Cl	O	3.218	0.984	153.9		[137]	
154	TEZTIC	Cl	O	3.00	0.917	168.7		[138]	
155	TINTEQ	Cl	O	3.033	0.927	162.3		[139]	
		Cl	O	3.002	0.918	168.3			
156	UGUKEM	Cl	O	3.264	0.998	166.7		[140]	
		Cl	Cl	3.309	0.945	160.2	150.8		Type 1
157	ULEMUU	Cl	O	3.227	0.987	168.8		[141]	
		Cl	O	3.212	0.982	170.4			
158	UNUYEI	Cl	O	3.234	0.989	172.6		[142]	
159	UVOKEW	Cl	O	3.152	0.964	175.4		[143]	
		Cl	Cl	3.484	0.995	174.8	88.0		
160	UVOKIA	Cl	O	3.020	0.924	164.3		[143]	Dianion, additional interaction with neutral LB
161	UVUWAK	Cl	O	2.985	0.913	155.5		[144]	Anionic functional group not in direct conjugation
162	UYEYIH	Cl	O	3.189	0.975	166.9		[145]	
		Cl	Cl	3.315	0.947	159.6	151.2		Type 1
163	UYOKOJ	Cl	O	3.00	0.917	171.1		[146]	
164	VUYWOC	Cl	O	3.205	0.980	163.3		[147]	
165	WIJBOJ	Cl	Cl	3.416	0.976	171.8	101.0	[148]	
		Cl	Cl	3.373	0.964	162.0	104.1		
		Cl	O	3.087	0.944	156.5			
166	WOGWUL	Cl	O	3.229	0.988	154.8		[149]	
		Cl	Cl	3.385	0.967	175.4	136.8		
		Cl	Cl	3.3775	0.965	168.5	136.2		
		Cl	Cl	3.4454	0.984	163.6	135.3		
167	WORPOK	Cl	O	3.097	0.947	176.1		[150]	
		Cl	Cl	3.280	0.937	172.4	77.7		
		Cl	Cl	3.374	0.964	144.7	98.2		

168		WORQEB	Cl	O	2.853	0.872	170.2		[150]	
169		WUPPAA	Cl	Cl	3.301	0.943	168.4	157.0	[151]	Type 1
			Cl	Cl	3.273	0.935	147.4	147.4		Type 1
			Cl	O	3.138	0.960	148.4			
170		XEMMIO	Cl	O	3.102	0.949	146.2		[152]	
171		XUNZEM	Cl	O	3.258	0.996	149.6		[153]	
			Cl	O	3.139	0.960	153.6			
172		YABVAY	Cl	O	3.087	0.944	162.2		[154]	
173		YAWDUX	Cl	O	3.103	0.949	156.4		[155]	
			Cl	Cl	3.378	0.965	143.1	137.8		Type 1
			Cl	Cl	3.378	0.965	143.1	137.9		Type 1
174		YUMROP	Cl	O	3.196	0.977	155.1		[156]	
175		YOTMUQ	Cl	O	3.257	0.996	149.9		[157]	
176		ZEWRUR	Cl	O	3.079	0.942	158.3		[158]	
			Cl	Cl	3.187	0.910	148.4	148.4		Type 1
177	1	AHARAC	Br	Br	3.654	0.988	149.7	128.7	[159]	Organometallic species
			Br	Br	3.529	0.954	157.3	105.5		
			Br	Br	3.654	0.988	149.7	128.7		
			Br	Br	3.529	0.954	157.3	105.5		
			Br	Br	3.534	0.955	170.2	85.5		
178	2	ATILEM	Br	Br	3.497	0.945	161.3	94.4	[160]	Organometallic species
179	3	BBZSUL	Br	O	3.365	0.999	169.9		[161]	Additional interaction with neutral LB
180	4	BIYGAT	Br	Br	3.397	0.918	155.8	155.8	[162]	Type 1
181	5	BUNDUL	Br	Br	3.435	0.928	150.4	150.4	[163]	Type 1
182	6	CAHLED	Br	O	3.190	0.947	146.4		[164]	dianion
183	7	CAHMAA	Br	Br	3.471	0.938	143.6	143.6	[165]	Type 1
184	8	CAQJAF	Br	Br	3.542	0.957	149.6	128.0	[166]	Organometallic species
			Br	Br	3.559	0.962	153.8	109.4		
			Br	Br	3.632	0.982	150.7	110.0		
185	9	COWRAK	Br	Br	3.624	0.979	147.4	146.1	[167]	Type 1
186	10	DBACCS10	Br	Br	3.645	0.985	162.9	121.2	[168]	
			Br	Br	3.690	0.997	158.8	130.7		
187	11	DOXLAG	Br	Br	3.676	0.994	154.8	88.8	[169]	
188	12	ERODOF	Br	O	3.317	0.948	144.8		[170]	
189	13	ESUNUD	Br	Br	3.567	0.964	148.3	137.1	[171]	Organometallic species, type 1
190	14	EXEJIB	Br	Br	3.6046	0.974	143.5	130.0	[172]	Type 1
191	15	FAGZOC	Br	Br	3.605	0.974	158.0	158.0	[173]	Type 1, dianion
192	16	FEKPAM	Br	Br	3.391	0.916	153.3	153.3	[174]	Type 1
193	17	GASHAJ	Br	O	2.979	0.884	172.6		[175]	

			Br	Br	3.575	0.966	168.1	149.0		
			Br	Br	3.524	0.951	143.5	127.5		
194	18	GIPNEB	Br	O	3.124	0.927	149.9		[176]	Organometallic species
195	19	GIPPAZ	Br	O	3.337	0.990	161.2		[176]	Organometallic species
196	20	GUNFUQ	Br	Br	3.577	0.967	162.3	137.5	[177]	Organometallic species
197	21	HUBXEJ	Br	O	3.250	0.964	175		[178]	dianion
			Br	O	3.227	0.958	157.8			
198	22	HONFOH	Br	O	3.225	0.957	158.8		[179]	
199	23	IBEGEE	Br	Br	3.532	0.955	165.8	119.2	[180]	Organometallic species
200	24	IBEQAK	Br	O	3.284	0.974	143.6		[181]	
201	25	IREQUT	Br	O	3.093	0.918	157.5		[182]	
202	26	JEPZIO	Br	Br	3.480	0.940	144.3	144.3	[183]	Type 1
203	27	JEQBIR	Br	Br	3.574	0.966	156.9	156.9	[183]	Type 1
204	28	JOVBII	Br	Br	3.626	0.980	143.9	143.9	[184]	Type 1
205	29	JOVCUV	Br	O	3.220	0.956	149.6		[184]	
206	30	KERTOT	Br	N	3.364	0.989	167.2		[185]	Negative functionality not in direct conjugation
207	31	KIDGOW	Br	O	3.253	0.965	154.7		[186]	
208	32	KUFTIO	Br	Br	3.562	0.963	147.5	138.5	[187]	Type 1
209	33	LABQIR	Br	Br	3.446	0.931	162.2	157.2	[188]	Type 1
210	34	LANLAO	Br	O	2.99	0.887	176.0		[189]	
211	35	LITXOC	Br	O	3.30	0.979	175.7		[190]	
212	36	LITYAP	Br	O	3.132	0.929	176.4		[190]	
			Br	O	3.302	0.980	170.7			
213	37	LITYIX	Br	O	3.254	0.966	171.7		[190]	
214	38	?	Br	O		0.92	179.7		[191]	
215	39	MAJTOH	Br	O	2.849	0.845	167.7		[192]	Additional interaction with cationic LB
			Br	O	2.849	0.845	171.2			
			Br	O	2.833	0.841	174.9			
			Br	O	2.808	0.833	177.5			
			Br	Br	3.578	0.967	159.8	102.5		
			Br	Br	3.636	0.983	146.2	92.7		
216	40	NAKKOB	Br	O	3.120	0.926	171.6		[193]	“halbe” negative Ladung
217	41	NAQFUI	Br	O	3.195	0.948	162.0		[194]	
218	42	NOGRUY	Br	O	2.994	0.888	162.4		[195]	
219	43	NUVPUP	Br	O	3.204	0.951	155.4		[196]	
220	44	OGERUO	Br	O	3.205	0.951	156.9		[197]	Additional interaction with neutral LB
			Br	Br	3.612	0.976	153.2	80.0		
221	45	OPIBAR	Br	Br	3.601	0.973	150.8	92.6	[198]	

222	46	POQPIX02	Br	Br	3.648	0.986	147.6	146.2	[199]	Type 1
223	47	QAJZUJ	Br	O	3.025	0.898	176.6		[200]	
224	48	QOTPEV	Br	O	3.118	0.925	154.5		[201]	
225	49	QIYVUD	Br	O	3.186	0.945	159.2		[202]	Organometallic species
226	50	REFCUC	Br	Br	3.421	0.924	166.6	120.0	[203]	
227	51	REYDOQ	Br	Br	3.570	0.965	174.1	174.1	[204]	Organometallic species, type 1
			Br	N	3.0956	0.910	168.1			
228	52	SORVIG	Br	O	3.325	0.987	146.7		[205]	
			Br	O	3.278	0.973	167.7			
229	53	SEYJAK	Br	Br	3.559	0.962	140.3	140.3	[206]	Type 1
			Br	O	3.231	0.959	154.4			
230	54	SORVUS	Br	O	3.065	0.909	169.1		[207]	dianion
231	55	SORWUT	Br	O	3.040	0.902	171.8		[205]	
			Br	Br	3.675	0.993	163.4	121.6		
232	56	SOXDOZ	Br	O	3.029	0.899	163.1		[208]	
233	57	SOYGAP	Br	O	3.359	0.997	145.8		[209]	
			Br	O	3.243	0.962	161.0			
234	58	TIJNOR	Br	O	3.029	0.899	170.9		[210]	
235	59	TUPKIZ	Br	I	3.637	0.930	172.4		[211]	Co-crystal, Pauling radius für I^-
236	60	UDASEA	Br	Br	3.329	0.890	170.3	154.9	[212]	Organometallic species
			Br	Br	3.317	0.896	168.0	149.4		
237	61	UMIDUR	Br	Br	3.693	0.998	152.8	123.8	[213]	Organometallic
			Br	Br	3.633	0.982	162.4	128.6		
			Br	Br	3.616	0.977	166.6	129.1		
			Br	Br	3.639	0.983	171.5	126.5		
			Br	Br	3.667	0.991	163.1	121.2		
238	62	UNUSAY	Br	Br	3.623	0.979	168.4		[214]	Type 1, additional interaction with neutral LB
239	63	UNUYAE	Br	O	2.934	0.871	172.1		[215]	
			Br	Br	3.592	0.971	154.7	108.9		
			Br	O	3.100	0.920	163.5			
240	64	UVUJEC	Br	Br	3.595	0.972	150.9	150.1	[216]	Type 1
241	65	UVUJUS	Br	O	3.302	0.98	159.1		[216]	dianion
242	66	UVUVAJ	Br	O	3.316	0.975	142.5		[144]	Anionic functionality not in direct conjugation
243	67	UYOKUP	Br	O	2.906	0.862	174.9		[217]	
244	68	VABTOJ	Br	O	3.111	0.923	173.3		[218]	dianion
245	69	VINQIU	Br	O	2.969	0.881	173.4		[219]	
246	70	VISVIE	Br	Br	3.661	0.990	147.5	147.5	[220]	Type 1
247	71	WEZLIZ	Br	F	3.318	0.999	140.2		[221]	
248	72	WUKMAQ	Br	Br	3.519	0.951	142.8	142.8	[222]	Type 1

249	73	XANJUT	Br	Br	3.649	0.986	144.7	144.7	[223]	Type 1
250	74	XAQREO	Br	Br	3.369	0.910	163.1	163.9	[224]	Type 1
251	75	XUBQOC	Br	Br	3.642	0.984	169.0	119.8	[225]	
			Br	Br	3.691	0.998	163.9	118.6		
252	76	YABLAP	Br	O	3.265	0.967	157.0		[226]	
			Br	O	3.102	0.920	173.5			
253	77	YEHWIT	Br	Br	3.628	0.981	170.9	104.2	[227]	
			Br	O	3.061	0.908	172.3			
254	78	YEHXAM	Br	O	3.126	0.928	165.3		[228]	
			Br	Br	3.637	0.983	152.2	130.9		
			Br	Br	3.582	0.968	173.2	119.2		
255	79	YEHYUH	Br	O	3.085	0.915	175.8		[229]	
			Br	Br	3.475	0.939	154.0	160.2		Type 1
256	80	YEHZAO	Br	O	3.174	0.942	169.6		[230]	
			Br	Br	3.696	0.999	143.6	121.0		
			Br	Br	3.619	0.978	153.0	70.7		
257	81	YEHZES	Br	O	3.041	0.902	166.4		[231]	
258	82	YIKPEP	Br	O	3.241	0.962	163.1		[232]	dianion
259	83	YIKPUF	Br	Br	3.620	0.978	150.9	82.3	[232]	
			Br	O	3.167	0.940	154.3			
260	84	YIKQEQ	Br	O	3.238	0.961	164.0		[232]	
261	85	YIKQIU	Br	O	3.273	0.971	148.0		[232]	
262	86	ZOPGES	Br	O	3.280	0.973	153.0		[233],[234]	
263	87	ZUHCIQ	Br	Br	3.6025	0.974	147.7	147.7	[235]	Type 1
264	1	ACACEO	I	I	3.807	0.961	151.8	151.8	[236]	Type 1
265	2	BIYFEW	I	O	3.053	0.872	164.0		[237]	
			I	F	3.348	0.970	153.3			
266	3	BIYFIA	I	O	2.993	0.855	160.3		[237]	
			I	O	2.976	0.850	164.3			
267	4	BIYFOG	I	O	2.872	0.824	164.0		[237]	
			I	O	2.884	0.824	166.3			
268	5	CELXEY	I	O	3.242	0.926	154.9		[236]	
269	6	CEZVIP	I	O	3.241	0.926	171.3		[238]	Additional interaction with neutral LB
270	7	DETCOX	I	I	3.738	0.944	157.6	157.6	[239]	Type 1
271	8	FUWFOV	I	I	3.920	0.938	169.0		[240]	Co-Crystal
272	9	HINCEK	I	O	3.103	0.887	150.3		[241]	Additional interaction with neutral LB
273	10	HINXEK	I	O	3.103	0.887	150.3		[241]	Additional interaction with neutral LB
274	11	MOKVAK	I	O	3.050	0.871	165.0		[242]	

275	12	NORQIX	I	O	2.934	0.838	174.3		[243]	
			I	O	2.864	0.818	169.1			
276	13	NORQOD	I	O	3.008	0.859	164.5		[243]	Additional interaction with neutral LB
			I	O	2.886	0.825	172.6			
277	14	OBUPOS	I	I	3.914	0.988	153.9	121.9	[244]	Type 1, Additional interaction with neutral LB
278	15	RIDFOA	I	I	3.748	0.947	147.8	147.8	[245]	Type 1, dianion
279	16	RIFOAO01	I	I	3.686	0.931	145.8	145.3	[245]	
			I	I	3.882	0.980	140.9	140.6		Type 1
280	17	SIQVEW	I	O	3.155	0.901	159.7		[246]	Additional interaction with neutral LB
			I	O	3.309	0.945	142.7			
			I	I	3.823	0.965	164.9	101.7		
281	18	TIVTIB	I	O	3.179	0.908	160.7		[247]	
			I	O	3.363	0.961	165.9			
282	19	TOHYAR	I	I	3.737	0.943	150.3		[248]	
283	20	TUBMUB	I	O	2.994	0.855	176.3		[249]	Additional interaction with cationic LB
284	21	TYRXEA10	I	I	3.919	0.990	169.4	73.1	[250]	
			I	I	3.694	0.93	147.6	140.2		Type 1
285	22	UDASOK	I	O	3.152	0.901	167.6		[212]	
			I	O	3.170	0.906	167.5			
			I	O	3.096	0.885	161.3			
			I	O	3.099	0.885	155.4			
			I	O	3.359	0.960	151.0			
			I	O	3.296	0.942	149.0			
286	23	VEXBUW	I	O	3.410	0.974	151.4		[251]	
287	24	WECDOZ	I	O	3.038	0.868	163.1		[252]	
288	25	WIKHAA	I	O	3.311	0.946	156.8		[253]	
			I	O	3.115	0.890	175.3			
289	26	WUJFUF	I	N	2.967	0.841	178.8		[254]	
290	27	WUJGEQ	I	N	3.027	0.858	172.8		[254]	
291	28	WUJGAM	I	I	3.331	0.797	177.5		[254]	Co-crystal
292	29	WUJGIU	I	Cl	3.022	0.797	173.6		[254]	Co-crystal
			I	Cl	3.051	0.805	175.2			Co-crystal
293	30	WUJGOA	I	I	3.337	0.806	177.6		[254]	Co-crystal
			I	I	3.416	0.817	171.8			Co-crystal
294	31	WUJGUG	I	N	2.796	0.792	172.5		[254]	
			I	N	2.855	0.809	171.6			
			I	N	2.887	0.818	170.0			
295	32	YEKYOE	I	O	3.461	0.989	140.4		[255]	

I	O	3.427	0.979	158.6
I	O	3.278	0.937	164.5

Inorganic Compounds

#	Ref.code	X	LB	$d_{X\cdots LB}$	R_{XB}	∡		Reference	Comment
4	AGONIV	Cl	O	3.065	0.937	154.4		[4]	Neutral LB
27	CECRAG	Cl	Cl	3.2596	0.931	166.7	162.0	[25]	Type 1, neutral LB
33	CEMFIM01	Cl	O	3.052	0.933	164.0		[34]	Neutral LB
48	DITJIA	Cl	Cl	3.469	0.991	139.6	148.4	[44]	neutral LB, type 1
		Cl	Cl	3.429	0.980	150.1	119.5		Neutral LB
		Cl	Cl	3.4315	0.980	151.1	137.7		Neutral LB
70	FOVCAV	Cl	H_2O	3.185	0.974	156.3		[65]	Neutral LB
160	UVOKIA	Cl	O	3.166	0.968	175.9		[143]	Neutral LB
296	AHOFAG	Br	Br	3.518	0.951	173.3		[256]	Inorganic
297	AHOHAI	Br	Br	3.342	0.903	179.0		[256]	inorganic
298	AZUDOP	Br	H_2O	2.978	0.883	169.8		[257],[258]	Neutral LB
179	BBZSUL	Br	OH_2	3.037	0.901	170.8		[161]	neutral LB
299	HIFXAY	Br	Br	3.567	0.964	165.1		[259]	inorganic
215	MAJTOH	Br	Br	3.636	0.983	146.2	92.7		Cationic LB
		Br	Br	3.578	0.967	159.8	102.5		Cationic LB
220	OGERUO	Br	HOMe	3.042	0.903	174.1		[197]	neutral LB
300	UNUROL	Br	O	3.172	0.941	172.7			neutral LB
238	UNUSAY	Br	O	3.122	0.926	166.8		[214]	neutral LB
301	ALAZUK	I	I	3.773	0.953	158.6		[260]	inorganic
269	CEZVIP	I	O	2.969	0.845	166.5		[238]	Neutral LB
302	EXAFEP	I	I	3.788	0.957	178.2	96.2	[261]	inorganic
303	GEJQIV	I	I	3.766	0.951	176.6		[262]	inorganic
272	HINCEK	I	O	2.970	0.849	169.2		[241]	neutral LB
273	HINXEK	I	O	2.970	0.848	169.2		[241]	Neutral LB
304	JIBHOR	I	I	3.770	0.952	180.0		[263]	inorganic
276	NORQOD	I	O	2.920	0.834	175.0		[243]	Neutral LB
277	OBUPOS	I	O	3.023	0.864	176.1		[244]	neutral LB
		I	I	3.835	0.968	156.5	75.7		Neutral LB
280	SIQVEW	I	O	3.197	0.913	156.1		[246]	Neutral LB
283	TUBMUB	I	N	3.109	0.881	175.9		[249]	Cationic LB

[1] Zaman, Tomura, Yamashita, *Organic letters* **2000**, *2*, 273.
[2] R. P. Sharma, A. Singh, P. Venugopalan, A. Rodríguez-Diéguez, J. M. Salas, *Polyhedron* **2012**, *47*, 173.
[3] T. Sugiyama, J. Meng, T. Matsuura, *Acta Cryst. C* **2002**, *58*, O242-6.
[4] M. Kaur, J. P. Jasinski, R. J. Butcher, H. S. Yathirajan, K. Byrappa, *Acta crystallographica. Section E, Structure reports online* **2013**, *69*, o1556-7.
[5] G. Smith, U. D. Wermuth, *Acta crystallographica. Section E, Structure reports online* **2013**, *69*, o1546.
[6] G. Mouchaham, M. Gualino, N. Roques, C. Duhayon, S. Brandès, J.-P. Sutter, *Cryst. Eng. Comm.* **2015**, *17*, 8906.
[7] J. Xia, X.-J. Wang, X.-J. Sun, H.-L. Zhu, D.-Q. Wang, *Zeitschrift für Kristallographie - New Crystal Structures* **2003**, *218*, 247.
[8] Z.-y. Chen, M.-x. Peng, *J. Chem. Crystallogr.* **2011**, *41*, 137.
[9] M. Ichikawa, *Acta Crystallogr B Struct Crystallogr Cryst Chem* **1972**, *28*, 755.
[10] K. Molčanov, V. Stilinović, A. Šantić, N. Maltar-Strmečki, D. Pajić, B. Kojić-Prodić, *Crystal Growth & Design* **2016**, *16*, 4777.
[11] K. Molčanov, C. Jelsch, B. Landeros, J. Hernández-Trujillo, E. Wenger, V. Stilinović, B. Kojić-Prodić, E. C. Escudero-Adán, *Crystal Growth & Design* **2019**, *19*, 391.

[12] J. Lü, L.-W. Han, J.-X. Lin, R. Cao, *Crystal Growth & Design* **2011**, *11*, 2035.
[13] K. Rajagopal, R. V. Krishnakumar, A. Mostad, S. Natarajan, *Acta Crystallogr. E Crystallogr. Commun.* **2003**, *59*, o31-o33.
[14] M. Akhtaruzzaman, M. Tomura, K. Takahashi, J.-I. Nishida, Y. Yamashita, *Supramolecular Chemistry* **2003**, *15*, 239.
[15] A. R. Kennedy, F. R. N. Waterson, *Acta Cryst. C* **2003**, *59*, o613-5.
[16] J. M. Sanders, A. O. Gómez, J. Mao, G. A. Meints, E. M. van Brussel, A. Burzynska, P. Kafarski, D. González-Pacanowska, E. Oldfield, *J. Med. Chem.* **2003**, *46*, 5171.
[17] B. T. Gowda, S. Foro, H. S. Spandana, *Acta crystallographica. Section E, Structure reports online* **2012**, *68*, m1368.
[18] D. Maspoch, D. Ruiz-Molina, K. Wurst, G. Vaughan, N. Domingo, J. Tejada, C. Rovira, J. Veciana, *Cryst. Eng. Comm.* **2004**, *6*, 573.
[19] I. Goldberg, *Journal of Inclusion Phenomena* **1984**, *1*, 349.
[20] S. L. Bekö, J. W. Bats, E. Alig, M. U. Schmidt, *J. Chem. Crystallogr.* **2013**, *43*, 655.
[21] S. A. Koch, M. Millar, *J. Am. Chem. Soc.* **1982**, *104*, 5255.
[22] Y. Tobu, R. Ikeda, T.-a. Nihei, K. Gotoh, H. Ishida, T. Asaji, *Phys. Chem. Chem. Phys.* **2012**, *14*, 12347.
[23] L. Julia, H. Suschitzky, J. C. Barnes, C. D. S. Tomlin, *J. Chem. Soc., Perkin Trans. 1* **1983**, 2507.
[24] C. H.L. Kennard, G. Smith, E. J. O'Reilly, *Inorg. Chim. Acta* **1983**, *77*, L181-L184.
[25] I. Sovago, L. H. Thomas, M. S. Adam, S. C. Capelli, C. C. Wilson, L. J. Farrugia, *Cryst. Eng. Comm.* **2016**, *18*, 5697.
[26] S. Takahashi, T. Jukurogi, T. Katagiri, K. Uneyama, *Cryst. Eng. Comm.* **2006**, *8*, 320.
[27] M. Konno, *Acta Cryst. C* **1984**, *40*, 236.
[28] G. J. Perpétuo, J. Janczak, *Acta Cryst. C* **2006**, *62*, o372-5.
[29] A. Rajam, P. T. Muthiah, R. J. Butcher, J. P. Jasinski, C. Glidewell, *Acta crystallographica. Section C, Structural chemistry* **2017**, *73*, 862.
[30] R. Roy, T. K. Adalder, P. Dastidar, *Chem. Asian J.* **2018**, *13*, 552.
[31] R. R. Holmes, R. O. Day, J. J. Harland, A. C. Sau, J. M. Holmes, *Organometallics* **1984**, *3*, 341.
[32] M. N. Peñas-Defrutos, C. Bartolomé, P. Espinet, *Organometallics* **2018**, *37*, 3533.
[33] K. Gotoh, R. Ishikawa, H. Ishida, *Acta crystallographica. Section E, Structure reports online* **2007**, *63*, o4518-o4518.
[34] D. Britton, M. K. Chantooni Jnr, W. J. Wang, I. M. Kolthoff, *Acta Cryst. C* **1984**, *40*, 1584.
[35] J. Maixner, J. Zachová, K. Huml, *Collect. Czech. Chem. Comm.* **1993**, *58*, 861.
[36] M. A. García-Monforte, I. Ara, A. Martín, B. Menjón, M. Tomás, P. J. Alonso, A. B. Arauzo, J. I. Martínez, C. Rillo, *Inorg. Chem.* **2014**, *53*, 12384.
[37] C. Miravilles, E. Molins, X. Solans, G. Germain, J. P. Declercq, *Journal of Inclusion Phenomena* **1985**, *3*, 27.
[38] B. Lou, S. R. Perumalla, C. C. Sun, *J. Mol. Struct.* **2015**, *1099*, 516.
[39] G. Smith, *Acta Crystallogr. E Crystallogr. Commun.* **2015**, *71*, 931.
[40] L. K. Mapp, S. J. Coles, S. Aitipamula, *Cryst. Eng. Comm.* **2017**, *19*, 2925.
[41] A. Elduque, Y. Garcés, F. J. Lahoz, J. A. López, L. A. Oro, T. Pinillos, C. Tejel, *Inorg. Chem. Commun.* **1999**, *2*, 414.
[42] H.-K. Fun, C. K. Quah, N. Boonnak, S. Chantrapromma, *Acta crystallographica. Section E, Structure reports online* **2013**, *69*, o1753-4.
[43] A. N. Chekhlov, A. I. Yurtanov, I. V. Martynov, *Dokl. Akad. Nauk SSSR (Russ.) (Proc. Nat. Acad. Sci. USSR)* **1985**, *282*, 323.
[44] M. Schmidtmann, C. C. Wilson, *Cryst. Eng. Comm.* **2008**, *10*, 177.
[45] E. Marfo-Owusu, A. Thompson, *X-ray Structure Analysis Online* **2014**, *30*, 45.
[46] I. Majerz, Z. Malarski, T. Lis, *Pol. J. Chem.* **1998**, *72*.
[47] R. R. Holmes, R. O. Day, A. C. Sau, C. A. Poutasse, J. M. Holmes, *Inorg. Chem.* **1986**, *25*, 607.
[48] S. Kawata, H. Kumagai, K. Adachi, S. Kitagawa, *J. Chem. Soc., Dalton Trans.* **2000**, 2409.
[49] S. V. Rosokha, J. Lu, T. Y. Rosokha, J. K. Kochi, *Phys. Chem. Chem. Phys.* **2009**, *11*, 324.
[50] A. N. Chekhlov, A. I. Yurtanov, I. V. Martynov, *Kristallografiya (Russ.) (Crystallogr. Rep.)* **1986**, *31*, 270.
[51] K. Gotoh, H. Ishida, *Acta crystallographica. Section E, Structure reports online* **2009**, *65*, o3060.
[52] H.-J. Lehmler, X. He, X. Li, M. W. Duffel, S. Parkin, *Chemosphere* **2013**, *93*, 1965.
[53] J. Zhang, S. Jin, L. Tao, B. Liu, D. Wang, *J. Mol. Struct.* **2014**, *1072*, 208.
[54] Z. P. Liang, *Acta crystallographica. Section E, Structure reports online* **2011**, *67*, o1357.
[55] L.-M. Li, Y.-F. Li, L. Liu, Z.-H. Zhang, *Acta crystallographica. Section E, Structure reports online* **2011**, *67*, m973.
[56] J. Palmucci, K. T. Mahmudov, M. F. C. Guedes da Silva, F. Marchetti, C. Pettinari, D. Petrelli, L. A. Vitali, L. Quassinti, M. Bramucci, G. Lupidi et al., *RSC Adv.* **2016**, *6*, 4237.
[57] R. Mattes, Dorau A., *Z. Naturforsch., B: Chem. Sci.* **1986**, *41*, 808.
[58] K. Gotoh, H. Ishida, *Acta Crystallogr. E Crystallogr. Commun.* **2017**, *73*, 1840.
[59] H. D. Becker, B. W. Skelton, A. H. White, *Aust. J. Chem.* **1987**, *40*, 625.
[60] G. Tosi, Bruni P., L. Cardellini, Stipa P., G. Bocelli, Rizzoli C., *Gazz. Chim. Ital.* **1989**, *119*, 399.
[61] R. Swinton Darious, P. Thomas Muthiah, F. Perdih, *Acta crystallographica. Section C, Structural chemistry* **2018**, *74*, 487.
[62] A. D. Morales, H. Novoa de Armas, L. Xuárez Marill, R. Pellón Comdom, S. García-Granda, *Acta Cryst. C* **1999**, *55*, IUC9900056.
[63] A. El-Dissouky, T. E. Khalil, H. A. Elbadawy, D. S. El-Sayed, A. A. Attia, S. Foro, *J. Mol. Struct.* **2020**, *1200*, 127066.
[64] M. Gallegos, S. Gil-Guerrero, A. Fernández-Alarcón, D. Bouzas-Ramos, J. Martín, C. Concellón, V. del Amo, J. M. Costa, R. Mendoza-Meroño, S. García-Granda et al., *Theor. Chem. Acc.* **2019**, *138*.
[65] J. Janczak, G. J. Perpétuo, *Acta Cryst. C* **2009**, *65*, o339-41.
[66] H.-L. Liu, S.-H. Guo, Y.-Y. Li, F.-F. Jian, *Acta Crystallogr. E Crystallogr. Commun.* **2009**, *65*, o1905.
[67] G. Smith, U. D. Wermuth, *J. Chem. Crystallogr.* **2010**, *40*, 207.
[68] J. Lee, H. Takahashi, Y. Matsui, T. Hori, *Acta crystallographica. Section E, Structure reports online* **2005**, *61*, o2403-o2405.
[69] U. K. Das, V. G. Puranik, P. Dastidar, *Crystal Growth & Design* **2012**, *12*, 5864.
[70] Z. Sun, J. Li, C. Ji, J. Sun, M. Hong, J. Luo, *J. Am. Chem. Soc.* **2017**, *139*, 15900.
[71] H. Zaher, A. E. Ashley, M. Irwin, A. L. Thompson, M. J. Gutmann, T. Krämer, D. O'Hare, *Chem. Commun.* **2013**, *49*, 9755.
[72] A. N. Chekhlov, I. V. Martynov, *Dokl. Akad. Nauk SSSR (Russ.) (Proc. Nat. Acad. Sci. USSR)* **1998**, *363*, 362.
[73] H. Ishida, S. Kashino, *Acta Cryst. C* **1999**, *55*, 1149.
[74] M. Atzori, F. Pop, P. Auban-Senzier, C. J. Gómez-García, E. Canadell, F. Artizzu, A. Serpe, P. Deplano, N. Avarvari, M. L. Mercuri, *Inorg. Chem.* **2014**, *53*, 7028.
[75] V. A. Trush, K. E. Gubina, V. M. Amirkhanov, J. Swiatek-Kozlowska, K. V. Domasevitch, *Polyhedron* **2005**, *24*, 1007.
[76] M. Odabaşogˇlu, O. Büyükgüngör, *Acta crystallographica. Section E, Structure reports online* **2006**, *62*, o739-o741.

[77] V. H. Rodrigues, A. Matos Beja, J. A. Paixão, M. M. R. R. Costa, *Acta Cryst. C* **2006**, *62*, o71-2.
[78] T. Tan, *J. Mol. Struct.* **2007**, *840*, 6.
[79] S. Houllemare-Druot, G. Coquerel, *J. Chem. Soc., Perkin Trans. 2* **1998**, 2211.
[80] G. Smith, U. D. Wermuth, *Acta crystallographica. Section E, Structure reports online* **2009**, *66*, o133.
[81] G. Smith, U. D. Wermuth, J. M. White, *Acta Cryst. C* **2008**, o532-6.
[82] K. Gotoh, H. Nagoshi, H. Ishida, *Acta crystallographica. Section E, Structure reports online* **2009**, o614.
[83] P. Su, X.-Y. Huang, X.-G. Meng, *Acta crystallographica. Section E, Structure reports online* **2008**, o2217-8.
[84] Zulkarnain, L. Miyan, A. Ahmad, M. Fazle Alam, H. Younus, *Journal of photochemistry and photobiology. B, Biology* **2017**, 195.
[85] T. Chu, Y. Hu, J. Wu, C. Zeng, Y. Yang, S. W. Ng, *Photochemical & photobiological sciences : Official journal of the European Photochemistry Association and the European Society for Photobiology* **2016**, 744.
[86] A. R. Kennedy, C. A. Morrison, N. E. B. Briggs, W. Arbuckle, *Crystal Growth & Design* **2011**, *11*, 1821.
[87] K. Rajagopal, R. D. Franklin, R. V. Krishnakumar, K. Ravikumar, S. Natarajan, *Acta crystallographica. Section E, Structure reports online* **2004**, *60*, o1355-o1357.
[88] D. R. Trivedi, A. Ballabh, P. Dastidar, B. Ganguly, *Chem. Eur. J.* **2004**, *10*, 5311.
[89] O. Kühl, S. Goutal, *Crystal Growth & Design* **2005**, *5*, 1875.
[90] C. Kingsbury, *CSD Communication (Private Communication)* **2017**.
[91] J.-X. Yuan, *Acta crystallographica. Section E, Structure reports online* **2005**, *61*, o3294-o3296.
[92] M. Chowdhury, B. M. Kariuki, *Crystal Growth & Design* **2006**, *6*, 774.
[93] T. Taga, T. Kobayashi, *Acta Cryst. C* **1990**, *46*, 1343.
[94] A. Ballabh, D. R. Trivedi, P. Dastidar, *Chem. Mater.* **2006**, *18*, 3795.
[95] R. Maskey, M. Schädler, C. Legler, L. Greb, *Angew. Chem. Int. Ed.* **2018**, 1717.
[96] Z. Dega-Szafran, A. Kania, M. Grundwald-Wyspiańska, M. Szafran, E. Tykarska, *J. Mol. Struct.* **1996**, *381*, 107.
[97] N. Bakewell, R. Thavarajah, M. Motevalli, T. S. Sheriff, *New J. Chem.* **2017**, *41*, 15411.
[98] K. Gotoh, R. Ishikawa, H. Ishida, *Acta crystallographica. Section E, Structure reports online* **2006**, *62*, o4738-o4740.
[99] I. Olovsson, H. Ptasiewicz-Bak, T. Gustafsson, I. Majerz, *Acta crystallographica. Section B, Structural science* **2002**, *58*, 627.
[100] J. Blignaut, A. Lemmerer, *Acta Crystallogr. E Crystallogr. Commun.* **2018**, 580.
[101] X. Shi, J. Luo, Z. Sun, S. Li, C. Ji, L. Li, L. Han, S. Zhang, D. Yuan, M. Hong, *Crystal Growth & Design* **2013**, *13*, 2081.
[102] C. F. Edwards, W. P. Griffith, A. J. P. White, D. J. Williams, *J. Chem. Soc., Dalton Trans.* **1992**, 957.
[103] J. A. Kanters, E. H. ter Horst, E. Grech, *Acta Cryst. C* **1992**, *48*, 1345.
[104] K. Gotoh, T. Asaji, H. Ishida, *Acta Cryst. C* **2010**, o114-8.
[105] L. F. Diniz, P. S. Carvalho, C. C. de Melo, J. Ellena, *Crystal Growth & Design* **2017**, *17*, 2622.
[106] H. Sugimoto, Y. Furukawa, M. Tarumizu, H. Miyake, K. Tanaka, H. Tsukube, *Eur. J. Inorg. Chem.* **2005**, *2005*, 3088.
[107] R. Basaran, S.-q. Dou, A. Weiss, *Struct Chem* **1993**, *4*, 219.
[108] S. Suresh, P. Pandi, R. M. Kumar, G. Chakkaravarthi, *IUCrData* **2017**, *2*.
[109] M. J. Ahlmark, J. J. Vepsäläinen, M. Ahlgrén, R. Niemi, H. Taipale, T. Järvinen, *Chem. Commun.* **2000**, 711.
[110] E. Bozkurt, I. Kartal, M. Odabaşoğlu, O. Büyükgüngör, *Acta crystallographica. Section E, Structure reports online* **2006**, *62*, o4258-o4260.
[111] M. J. Horner, K. T. Holman, M. D. Ward, *J. Am. Chem. Soc.* **2007**, *129*, 14640.
[112] P.-W. Zhang, T.-Y. Zhang, L. Zhang, Y. Deng, *Acta Crystallogr. E Crystallogr. Commun.* **2008**, *64*, o641.
[113] K. S. S. Babu, G. Peramaiyan, M. NizamMohideen, R. Mohan, *Acta crystallographica. Section E, Structure reports online* **2014**, *70*, o391-2.
[114] S. Sen, M. Kumar Saha, S. Mitra*, A. J. Edwards, W. Clegg*, *Polyhedron* **2000**, *19*, 1881.
[115] G. Smith, U. D. Wermuth, J. M. White, *Acta Cryst. C* **2009**, *65*, o103-7.
[116] K. Hafner, T. M. Klapötke, P. C. Schmid, J. Stierstorfer, *Eur. J. Inorg. Chem.* **2015**, *2015*, 2794.
[117] J. Galloy, J.-P. Putzeys, G. Germain, M. van Meerssche, *Acta Crystallogr B Struct Crystallogr Cryst Chem* **1976**, *32*, 2718.
[118] H. Ishida, B. Rahman, S. Kashino, *Acta Cryst. C* **2001**, *57*, 1450.
[119] S. Banerjee, Ankur, A. P. Andrews, B. Varghese, A. Venugopal, *Dalton transactions (Cambridge, England : 2003)* **2019**, 7313.
[120] S. Ebenezer, P. T. Muthiah, *Crystal Growth & Design* **2012**, *12*, 3766.
[121] A. Czylkowska, R. Kruszyski, A. Kaczmarek, M. Markiewicz, *Zh. Strukt. Khim. (Russ.) (J. Struct. Chem.)* **2012**, *53*, 946.
[122] S. Sen, S. Mitra, P. Kundu, M. K. Saha, C. Krüger, J. Bruckmann, *Polyhedron* **1997**, *16*, 2475.
[123] M. G. Amiri, A. Morsali, *Z. Anorg. Allg. Chem.* **2006**, *632*, 1419.
[124] J. H. Buttery, Effendy, P. C. Junk, S. Mutrofin, B. W. Skelton, C. R. Whitaker, A. H. White, *Z. Anorg. Allg. Chem.* **2006**, *632*, 1326.
[125] H. Akutsu, J.-i. Yamada, S.'i. Nakatsuji, S. S. Turner, *Solid State Communications* **2007**, *144*, 144.
[126] Z. Rzączyńska, R. Mrozek, M. Sikorska-Iwan, T. Głowiak, *Journal of Coordination Chemistry* **2000**, *49*, 189.
[127] G. Smith, U. D. Wermuth, *Acta Cryst. C* **2010**, *66*, o374-80.
[128] M. V. N. de Souza, R. A. Howie, E. R. T. Tiekink, J. L. Wardell, S. M. S. V. Wardell, *Acta Crystallogr. E Crystallogr. Commun.* **2009**, *65*, o3204-5.
[129] C. Sporer, I. Ratera, K. Wurst, J. Vidal-Gancedo, D. Ruiz-Molina, C. Rovira, J. Veciana, *Arkivoc* **2005**, *6*, 104.
[130] P. Sanphui, G. Bolla, A. Nangia, *Crystal Growth & Design* **2012**, *12*, 2023.
[131] K. Thanigaimani, A. Farhadikoutenaei, N. C. Khalib, S. Arshad, I. A. Razak, *Acta crystallographica. Section E, Structure reports online* **2012**, *68*, o3195.
[132] I. Majerz, Z. Malarski, T. Lis, *J. Chem. Crystallogr.* **1990**, *20*, 187.
[133] G. Smith, U. D. Wermuth, J. M. White, *Acta crystallographica. Section E, Structure reports online* **2007**, *63*, o4276-o4277.
[134] X. Wang, *CSD Communication (Private Communication)* **2019**.
[135] J. C. Dewan, *Acta Crystallogr B Struct Crystallogr Cryst Chem* **1980**, *36*, 1935.
[136] D. Wallace, J. Reglinski, M. K. Taylor, A. R. Kennedy, *Acta crystallographica. Section E, Structure reports online* **2006**, *62*, m339-m341.
[137] R. Roy, P. Dastidar, *Chem. Eur. J.* **2017**, *23*, 15623.
[138] T. Raghavalu, K. S. Kumar, G. R. Kumar, S. Gokul Raj, R. Mohan, *Acta Crystallogr. E Crystallogr. Commun.* **2007**, *63*, o1706-o1707.
[139] H.-Y. Liu, J.-C. Ma, J. Yang, *Acta crystallographica. Section E, Structure reports online* **2007**, *63*, m2734-m2735.

[140] M.-L. Hu, M.-D. Ye, S. M. Zain, S. W. Ng, *Acta crystallographica. Section E, Structure reports online* **2002**, *58*, o1005-o1007.
[141] J. Li, *Acta crystallographica. Section E, Structure reports online* **2011**, *67*, o605.
[142] J. Li, *Acta crystallographica. Section E, Structure reports online* **2011**, *67*, o901.
[143] P. G. Waddell, J. O. S. Hulse, J. M. Cole, *Acta Cryst. C* **2011**, *67*, o255-8.
[144] H. Akutsu, T. Sasai, J. Yamada, S. Nakatsuji, S. S. Turner, *Physica B: Condensed Matter* **2010**, *405*, S2-S5.
[145] P. V. Dhanaraj, N. P. Rajesh, G. Vinitha, G. Bhagavannarayana, *Materials Research Bulletin* **2011**, *46*, 726.
[146] T. Kimoto, N. Shiota, T. Kinuta, T. Sato, N. Tajima, H. Tokutome, R. Kuroda, M. Fujiki, Y. Matsubara, Y. Imai, *Tetrahedron* **2011**, *67*, 7775.
[147] G. Smith, U. D. Wermuth, *Acta crystallographica. Section E, Structure reports online* **2010**, *66*, o1254.
[148] P. Rekha, P. Jayaprakash, G. Rajasekar, R. Mohan Kumar, G. Vinitha, R. Kanagadurai, *J. Mol. Struct.* **2019**, *1177*, 579.
[149] K. Gotoh, H. Ishida, *Acta crystallographica. Section E, Structure reports online* **2008**, *64*, o2095.
[150] Y. Hu, Z. Li, Y. Zhao, Y. Yang, F. Liu, L. Wang, *RSC Adv.* **2015**, *5*, 10275.
[151] A. A. Nikiforov, A. V. Eremin, A. N. Belyaev, V. V. Gurzhiy, *CSD Communication (Private Communication)* **2015**.
[152] M. Oruganti, S. K. Nechipadappu, P. A. Khade, D. R. Trivedi, *ACS omega* **2017**, *2*, 7146.
[153] H.-L. Cai, T. Zhang, L.-Z. Chen, R.-G. Xiong, *J. Mater. Chem.* **2010**, *20*, 1868.
[154] R. Basaran, S.-q. Dou, A. Weiss, *Ber. Bunsenges. Phys. Chem.* **1991**, *95*, 46.
[155] A. S. Dayananda, H. S. Yathirajan, U. Flörke, *Acta crystallographica. Section E, Structure reports online* **2012**, *68*, o968.
[156] S. Jin, Y. Zhao, B. Liu, X. Jin, H. Zhang, X. Wen, H. Liu, L. Jin, D. Wang, *J. Mol. Struct.* **2015**, *1099*, 601.
[157] Y.-H. Yu, K. Qian, *Acta crystallographica. Section E, Structure reports online* **2009**, *65*, o1278.
[158] A. Jagadesan, N. Sivakumar, S. Arjunan, G. Chakkaravarthi, *CSD Communication (Private Communication)* **2018**.
[159] M. Merkel, F. K. Müller, B. Krebs, *Inorg. Chim. Acta* **2002**, *337*, 308.
[160] M. Merkel, D. Schnieders, S. M. Baldeau, B. Krebs, *Berichte der deutschen chemischen Gesellschaft* **2004**, *2004*, 783.
[161] J.-O. Lundgren, *Acta Crystallogr B Struct Crystallogr Cryst Chem* **1972**, *28*, 475.
[162] A. Takemura, L. J. McAllister, S. Hart, N. E. Pridmore, P. B. Karadakov, A. C. Whitwood, D. W. Bruce, *Chem. Eur. J.* **2014**, *20*, 6721.
[163] C. B. Aakeröy, C. L. Spartz, S. Dembowski, S. Dwyre, J. Desper, *IUCrJ* **2015**, *2*, 498.
[164] M. B. Zaman, M. Tomura, Y. Yamashita, *J. Org. Chem.* **2001**, *66*, 5987.
[165] M. B. Zaman, M. Tomura, Y. Yamashita, *J. Org. Chem.* **2001**, *66*, 5987.
[166] H.-C. Chang, T. Ishii, M. Kondo, S. Kitagawa, *J. Chem. Soc., Dalton Trans.* **1999**, 2467.
[167] S. Horiuchi, S. Ishibashi, K. Kobayashi, R. Kumai, *RSC Adv.* **2019**, *9*, 39662.
[168] T. Głowiak, V. Videnova, J. Baran, H. Ratajczak, *Acta Crystallogr B Struct Crystallogr Cryst Chem* **1980**, *36*, 2412.
[169] L. Mo, S. Jin, W. Zhang, J. Guo, H. Liu, D. Wang, *J. Mol. Struct.* **2020**, *1205*, 127538.
[170] J. Li, *Acta crystallographica. Section E, Structure reports online* **2011**, *67*, o1356.
[171] A. Panja, N. C. Jana, A. Bauzá, A. Frontera, C. Mathonière, *Inorg. Chem.* **2016**, *55*, 8331.
[172] J. Lü, L.-W. Han, J.-X. Lin, R. Cao, *Crystal Growth & Design* **2011**, *11*, 3551.
[173] C. Robl, A. Weiss, *Z. Naturforsch.,B: Chem. Sci.* **1986**, *41*, 1495.
[174] v. d. Cherepinskii-Malov, Y. T. Struchkov, L. I. Gurarii, E. T. Mukmenev, B. A. Arbuzov, *Russ. J. Gen. Chem.* **1985**, *55*, 2457.
[175] I. Majerz, Z. Malarski, T. Lis, *Bull. Pol. Acad. Sci., Chem.* **1987**, *35*, 187.
[176] S. D. Kurbah, M. Asthana, I. Syiemlieh, A. A. Lywait, M. Longchar, R. A. Lal, *J. Organomet. Chem.* **2018**, *876*, 10.
[177] H. C. Chang, H. Miyasaka, S. Kitagawa, *Inorg. Chem.* **2001**, *40*, 146.
[178] S. K. Dey, R. Saha, S. Singha, S. Biswas, A. Layek, S. Middya, P. P. Ray, D. Bandhyopadhyay, S. Kumar, *Spectrochimica acta. Part A, Molecular and biomolecular spectroscopy* **2015**, *144*, 43.
[179] X. Gao, S. Jin, L. Jin, X. Ye, L. Zheng, J. Li, B. Jin, D. Wang, *J. Mol. Struct.* **2014**, *1075*, 384.
[180] A. Panja, N. C. Jana, M. Patra, P. Brandão, C. E. Moore, D. M. Eichhorn, A. Frontera, *J. Mol. Catal. A-Chem.* **2016**, *412*, 56.
[181] P. G. Jones, E.-M. Zerbe, *CSD Communication (Private Communication)* **2016**.
[182] J. M. Spaniol, K. A. Wheeler, *RSC Adv.* **2016**, *6*, 64921.
[183] A. Ballabh, D. R. Trivedi, P. Dastidar, *Chem. Mater.* **2006**, *18*, 3795.
[184] D. Choudhari, D. Chakravarty, D. N. Lande, S. Parveen, S. P. Gejji, K. M. Kodam, S. Salunke-Gawali, *Struct Chem* **2019**, *30*, 2257.
[185] Y. S. Kayukov, S. V. Karpov, A. A. Grigor'ev, O. E. Nasakin, V. A. Tafeenko, K. A. Lyssenko, A. V. Shapovalov, E. A. Varaksina, *Dalton transactions (Cambridge, England : 2003)* **2017**, *46*, 16925.
[186] J. Blignaut, A. Lemmerer, *Acta Crystallogr. E Crystallogr. Commun.* **2018**, *74*, 580.
[187] I. Willner, Y. Eichen, M. Rabinovitz, R. Hoffman, S. Cohen, *J. Am. Chem. Soc.* **1992**, *114*, 637.
[188] Blaschette A., Jones P. G., Lozano V., M. Freytag, *CSD Communication (Private Communication)*.
[189] B. Jindawong, S. Chantrapromma, H.-K. Fun, X.-L. Yu, C. Karalai, *Acta crystallographica. Section E, Structure reports online* **2005**, *61*, o1340-o1342.
[190] M. J. Horner, K. T. Holman, M. D. Ward, *J. Am. Chem. Soc.* **2007**, *129*, 14640.
[191] M. J. Horner, K. T. Holman, M. D. Ward, *Angew. Chem. Int. Ed.* **2001**, *40*, 4045.
[192] P. G. Jones, V. Lozano, *Acta Cryst. C* **2004**, *60*, o876-8.
[193] J. Xiong, Y. Guo, Z.-Y. Li, Z. M. Jin, *J. Chem. Crystallogr.* **2010**, *40*, 884.
[194] D. Saftić, B. Žinić, A. Višnjevac, *Tetrahedron* **2012**, *68*, 1062.
[195] D. C. Horton, D. VanDerveer, J. Krzystek, J. Telser, T. Pittman, D. C. Crans, A. A. Holder, *Inorg. Chim. Acta* **2014**, *420*, 112.
[196] J. Costante, N. Ehlinger, M. Perrin, Collet A., *Enantiomer* **1996**, *1*, 377.
[197] Z.-P. Liang, *Acta Crystallogr. E Crystallogr. Commun.* **2008**, *64*, o2416.
[198] E. C. Escudero-Adán, M. Martínez Belmonte, J. Benet-Buchholz, A. W. Kleij, *Organic letters* **2010**, *12*, 4592.
[199] Y. Yamamura, E. Saito, H. Saitoh, N. Hoshino, K. Saito, *Chem. Lett.* **2012**, *41*, 119.
[200] H. K. Fun, S. Chantrapromma, P. Jansrisewangwong, *Acta crystallographica. Section E, Structure reports online* **2010**, *67*, o105-6.
[201] S. Chantrapromma, T. Kobkeatthawin, H.-K. Fun, *Acta crystallographica. Section E, Structure reports online* **2009**, *65*, o950-1.
[202] X. Zhang, X. Jiang, Y. Li, Z. Lin, G. Zhang, Y. Wu, *Chemical Physics Letters* **2015**, *641*, 141.
[203] A. Panja, *Inorg. Chem. Commun.* **2012**, *24*, 140.
[204] A. Panja, *RSC Adv.* **2013**, *3*, 4954.
[205] L. Wang, Y. Hu, W. Xu, Y. Pang, F. Liu, Y. Yang, *RSC Adv* **2014**, *4*, 56816.

[206] M. Rok, G. Bator, W. Sawka-Dobrowolska, P. Durlak, M. Moskwa, W. Medycki, L. Sobczyk, M. Zamponi, *Cryst. Eng. Comm.* **2018**, *20*, 2016.
[207] L. Wang, Y. Hu, W. Xu, Y. Pang, F. Liu, Y. Yang, *RSC Adv* **2014**, *4*, 56816.
[208] S. Chantrapromma, K. Chanawanno, H.-K. Fun, *Acta crystallographica. Section E, Structure reports online* **2009**, *65*, o1144-5.
[209] L. H. Thomas, B. Boyle, L. A. Clive, A. Collins, L. D. Currie, M. Gogol, C. Hastings, A. O. F. Jones, J. L. Kennedy, G. B. Kerr et al., *Acta crystallographica. Section E, Structure reports online* **2009**, *65*, o1218.
[210] S. Chantrapromma, P. Ruanwas, B. Jindawong, H.-K. Fun, *Acta crystallographica. Section E, Structure reports online* **2013**, *69*, o1623-4.
[211] C. Surasit, S. Chantrapromma, K. Chanawanno, H.-K. Fun, *Acta crystallographica. Section E, Structure reports online* **2010**, *66*, o1372-3.
[212] P. G. Jones, C. Wolper, *CSD Communication (Private Communication)* **2016**.
[213] N. C. Jana, P. Brandão, A. Panja, *Journal of inorganic biochemistry* **2016**, *159*, 96.
[214] J. Li, *Acta crystallographica. Section E, Structure reports online* **2011**, *67*, o869.
[215] J. Li, *Acta crystallographica. Section E, Structure reports online* **2011**, *67*, o900.
[216] K. Łuczyńska, K. Drużbicki, K. Lyczko, J. C. Dobrowolski, *Crystal Growth & Design* **2016**, *16*, 6069.
[217] T. Kimoto, N. Shiota, T. Kinuta, T. Sato, N. Tajima, H. Tokutome, R. Kuroda, M. Fujiki, Y. Matsubara, Y. Imai, *Tetrahedron* **2011**, *67*, 7775.
[218] K.-L. Zhang, H. Huang, S. W. Ng, *Acta crystallographica. Section E, Structure reports online* **2010**, *66*, o2919.
[219] M. M. Najafpour, D. M. Boghaei, V. McKee, *Main Group Chemistry* **2009**, *8*, 11.
[220] S. Chakraborty, S. Ganguly, G. R. Desiraju, *Cryst. Eng. Comm.* **2014**, *16*, 4732.
[221] L. L. Liu, L. L. Cao, D. Zhu, J. Zhou, D. W. Stephan, *Chem. Commun.* **2018**, *54*, 7431.
[222] G. Wojciechowski, A. Katrusiak, B. Brzezinski, *J. Mol. Struct.* **2002**, *604*, 279.
[223] A. Kobayashi, M.-a. Dosen, M. Chang, K. Nakajima, S.-i. Noro, M. Kato, *J. Am. Chem. Soc.* **2010**, *132*, 15286.
[224] K. Molčanov, B. Kojić-Prodić, *Acta crystallographica. Section B, Structural science* **2012**, *68*, 57.
[225] X. X. Wang, Y. J. Ma, H. H. Li, G. H. Cui, *Transition Met. Chem. (Transition Metal Chemistry)* **2015**, *40*, 99.
[226] V. Lozano, O. Moers, P. G. Jones, A. Blaschette, *Z.Naturforsch., B: Chem. Sci.* **2004**, *59*, 661.
[227] J. Li, *Zeitschrift für Kristallographie - New Crystal Structures* **2012**, *227*, 325.
[228] J. Li, *Zeitschrift für Kristallographie - New Crystal Structures* **2012**, *227*, 333.
[229] J. Li, *Zeitschrift für Kristallographie - New Crystal Structures* **2012**, *227*, 355.
[230] J. Li, *Zeitschrift für Kristallographie - New Crystal Structures* **2012**, *227*, 357.
[231] J. Li, *Zeitschrift für Kristallographie - New Crystal Structures* **2012**, *227*, 359.
[232] L. H. Thomas, M. S. Adam, A. O'Neill, C. C. Wilson, *Acta Cryst. C* **2013**, *69*, 1279.
[233] A. Piecha-Bisiorek, G. Bator, W. Sawka-Dobrowolska, L. Sobczyk, M. Rok, W. Medycki, G. J. Schneider, *J. Phys. Chem. A* **2014**, *118*, 7159.
[234] K. Łuczyńska, K. Drużbicki, K. Lyczko, W. Starosta, *Vibrational Spectroscopy* **2014**, *75*, 26.
[235] H. Zhang, S. Jin, X. Wen, B. Liu, Y. Fang, Y. Zhang, D. Wang, *J. Mol. Struct.* **2015**, *1092*, 211.
[236] R. Montis, M. B. Hursthouse, *Cryst. Eng. Comm.* **2012**, *14*, 7466.
[237] A. Takemura, L. J. McAllister, S. Hart, N. E. Pridmore, P. B. Karadakov, A. C. Whitwood, D. W. Bruce, *Chem. Eur. J.* **2014**, *20*, 6721.
[238] G. Zhao, C. He, W. Zhou, J. P. Hooper, G. H. Imler, D. A. Parrish, J.'n. M. Shreeve, *Inorg. Chem.* **2018**, *57*, 8673.
[239] P. Liebing, F. Oehler, M. Wagner, P. F. Tripet, A. Togni, *Organometallics* **2018**, *37*, 570.
[240] T. Maxson, A. S. Jalilov, M. Zeller, S. V. Rosokha, *Angew. Chem.* **2020**, *132*, 17350; *Angew. Chem. Int. Ed.*, **2020**.
[241] J. L. Andrews, E. Pearson, D. S. Yufit, J. W. Steed, K. Edkins, *Crystal Growth & Design* **2018**, *18*, 7690.
[242] A. Lemmerer, *Acta Cryst. C* **2008**, *64*, o626-9.
[243] S. A. Adonin, M. A. Bondarenko, A. S. Novikov, P. A. Abramov, M. N. Sokolov, V. P. Fedin, *Cryst. Eng. Comm.* **2019**, *21*, 6666.
[244] S. Malathy, P. T. Muthiah, *Acta Crystallogr. E Crystallogr. Commun.* **2011**, *67*, o3241-2.
[245] S. Horiuchi, R. Kumai, Y. Tokura, *Angewandte Chemie (International ed. in English)* **2007**, *46*, 3497.
[246] G. Bousrez, P. C. Andrews, P. C. Junk, D. T. Thielemann, J. Wang, *Aust. J. Chem.* **2018**, *71*, 939.
[247] L. E. Tønnesen, B. F. Pedersen, J. Klaveness, W. T. Robinson, B. R. Wood, W. Errington, C. E. Olsen, C. N. Rosendahl, M. Haugg, N. Trabesinger-Rüf et al., *Acta Chem. Scand.* **1996**, *50*, 603.
[248] A. Lemmerer, S. A. Bourne, M. A. Fernandes, *Cryst. Eng. Comm.* **2008**, *10*, 1750.
[249] W. Yu, M. Zhang, K. Liu, Y. Yang, L. Wang, *Chem. Res. Chin. Univ.* **2019**, *35*, 806.
[250] V. Cody, *Acta Crystallogr B Struct Crystallogr Cryst Chem* **1981**, *37*, 1685.
[251] S. Friedrichs, P. G. Jones, *Z.Naturforsch., B: Chem. Sci.* **2006**, *61*, 1391.
[252] T. Sasaki, I. Hisaki, S. Tsuzuki, N. Tohnai, M. Miyata, *Cryst. Eng. Comm.* **2012**, *14*, 5749.
[253] E.-M. Zerbe, M. Freytag, P. G. Jones, A. Blaschette, *Z. Naturforsch., B: Chem. Sci.* **2007**, *62*, 1157.
[254] J. M. Holthoff, E. Engelage, R. Weiss, S. M. Huber, *Angew. Chem. Int. Ed.* **2020**, *59*, 11150; *Angew. Chem.*, **2020**, *132*, 11244–11251.
[255] K. Fucke, M. J. G. Peach, J. A. K. Howard, J. W. Steed, *Chem. Commun.* **2012**, *48*, 9822.
[256] A. N. Chernyshev, M. V. Chernysheva, P. Hirva, V. Y. Kukushkin, M. Haukka, *Dalton Trans.* **2015**, *44*, 14523.
[257] S. K. Dey, R. Saha, S. Biswas, A. Layek, S. Middya, I. M. Steele, M. Fleck, P. P. Ray, S. Kumar, *Crystal Growth & Design* **2014**, *14*, 207.
[258] H. Kumagai, S. Kawata, *Acta crystallographica. Section E, Structure reports online* **2011**, *67*, o2636.
[259] S. A. Adonin, M. A. Bondarenko, P. A. Abramov, A. S. Novikov, P. E. Plyusnin, M. N. Sokolov, V. P. Fedin, *Chem. Eur. J.* **2018**.
[260] C. Taouss, P. G. Jones, *Zeitschrift für Naturforschung B* **2016**, *71*, 249.
[261] J. S. Ovens, A. R. Geisheimer, A. A. Bokov, Z.-G. Ye, D. B. Leznoff, *Inorg. Chem.* **2010**, *49*, 9609.
[262] P. H. Svensson, J. Rosdahl, L. Kloo, *Chem. Eur. J.* **1999**, *5*, 305.
[263] J. Fábry, B. A. Maximov, *Acta Cryst. C* **1991**, *47*, 51.
